# A multi-stage association study of plasma cytokines identifies osteopontin as a biomarker for acute coronary syndrome risk and severity

**DOI:** 10.1038/s41598-019-41577-4

**Published:** 2019-03-26

**Authors:** Kuai Yu, Binyao Yang, Haijing Jiang, Jun Li, Kai Yan, Xuezhen Liu, Lue Zhou, Handong Yang, Xiulou Li, Xinwen Min, Ce Zhang, Xiaoting Luo, Wenhua Mei, Shunchang Sun, Liyun Zhang, Xiang Cheng, Meian He, Xiaomin Zhang, An Pan, Frank B. Hu, Tangchun Wu

**Affiliations:** 1 Department of Occupational and Environmental Health, Key Laboratory of Environment and Health, Ministry of Education and State Key Laboratory of Environmental Health (Incubating), School of Public Health, Tongji Medical College, Huazhong University of Science and Technology, 13 Hangkong Rd, Wuhan, 430030 Hubei China; 2 Department of Epidemiology and Biostatistics, School of Public Health, Tongji Medical College, Huazhong University of Science and Technology, 13 Hangkong Rd, Wuhan, 430030 Hubei China; 3 The Department of Cardiovascular Diseases, Dongfeng Central Hospital, Hubei University of Medicine, Shiyan, 442000 China; 4 Department of Cardiology, People’s Hospital of Zhuhai, Zhuhai, Guangdong, China; 5 Department of Cardiology, Bao’an Hospital, Shenzhen, Guangdong, China; 6 Department of Cardiology, Wuhan Central Hospital, Wuhan, Hubei China; 7 Department of Cardiology, Wuhan Union Hospital, Wuhan, Hubei China; 8 The Department of Nutrition and Department of Epidemiology, Harvard T.H. Chan School of Public Health, Boston, Massachusetts, 02115 United States; 9 Department of Central Laboratory, the 5th Affiliated Hospital of Guangzhou Medical University, Guangzhou, China

## Abstract

Cytokines play a critical role in the pathogenesis and development of cardiovascular diseases. However, data linking cytokines to risk and severity of acute coronary syndrome (ACS) are still limited. We measured plasma profile of 280 cytokines using a quantitative protein microarray in 12 ACS patients and 16 healthy controls, and identified 15 differentially expressed cytokines for ACS. Osteopontin, chemokine ligand 23, brain derived neurotrophic factor and C-reactive protein (CRP) were further validated using immunoassay in two independent case-control studies with a total of 210 ACS patients and 210 controls. We further examined their relations with incident ACS among 318 case-control pairs nested within the Dongfeng-Tongji cohort, and found plasma osteopontin and CRP concentrations were associated with incident ACS, and the multivariable-adjusted odds ratio (95% confidence interval) was 1.29 (1.06–1.57) per 1-SD increase for osteopontin and 1.30 (1.02–1.66) for CRP, respectively. Higher levels of circulating osteopontin were also correlated with higher severity of ACS, and earlier ACS onset time. Adding osteopontin alone or in combination with CRP modestly improved the predictive ability of ACS beyond the Framingham risk scores. Our findings suggested that osteopontin might be a biomarker for incident ACS, using osteopontin adds moderately to traditional cardiovascular risk factors.

## Introduction

Coronary heart disease (CHD) remains a leading cause of mortality and morbidity worldwide, claiming approximately 7 million deaths each year^1^, and Acute coronary syndrome (ACS) reflects the most urgent and severest clinical condition of CHD^2^. The pathophysiology of ACS remains to be fully understood but chronic inflammation has been widely considered as a potential contributor^3^. Several inflammatory biomarkers have been reported to be associated with ACS risk, and most of them were selected based on their presumed pathophysiological roles in ACS. However, a large degree of uncertainty remains regarding early detection and risk discrimination of ACS, and new biomarkers for better ACS risk prediction are urgently needed.

Cytokines are pleiotropic proteins mainly released from immune cells, which can act in concert with specific receptors or inhibitors to regulate inflammation^4,5^. Previous studies^6,7^ have identified several cytokines like interleukin-6 and C-reactive protein (CRP) as potential biomarkers for atherosclerosis, myocardial infarction, or main biological pathways involved in those cardiovascular conditions. However, few studies have used high-throughput methods to screen for a large number of cytokines as potential biomarkers for ACS.

In this multi-stage study, using a protein microarray which facilitate standardized comparison of the observed associations across cytokines, we aimed to identify novel cytokines associated with ACS, and to examine the predictive value of identified cytokines beyond traditional ACS risk factors.

## Results

### Basic characteristics of the study population

Table 1 showed the demographic characteristics of ACS patients and controls in each study set. Compared with controls, ACS patients were more likely to have hypertension, diabetes, and to take anti-hypertensive and lipid-lowing medication across all study sets (all *P* < 0.05).

**Table 1:** Demographic and Clinical Characteristics of the Study Populations.

Variables	Discovery stage			Validation set 1			Validation set 2			Nested case-control study *		
	Controls (n = 16)	Cases (n = 12)	*P*	Controls (n = 107)	Cases (n = 107)	*P*	Controls (n = 103)	Cases (n = 103)	*P*	Controls (n = 318)	Cases (n = 318)	*P*
Age, years	50.4 ± 4.9	50.8 ± 7.6	0.848	53.6 ± 7.0	55.1 ± 8.3	0.121	49.9 ± 10.3	52.1 ± 10.6	0.226	67.0 ± 7.1	67.1 ± 7.3	0.983
Man, N (%)	12 (75.0)	9 (75.0)	1.000	84 (78.5)	84 (78.5)	1.000	85 (82.5)	85 (82.5)	0.106	170 (53.5)	170 (53.5)	1.000
BMI, kg/m^2^	25.9 ± 2.6	25.0 ± 2.9	0.915	24.0 ± 2.6	24.4 ± 2.6	0.213	24.5 ± 3.1	23.9 ± 2.5	0.096	24.7 ± 3.4	24.7 ± 3.4	0.879
Current smokers, N (%)	6 (37.5)	6 (50.0)	0.702	57 (53.3)	56 (52.3)	0.509	48 (46.6)	47 (45.6)	0.584	76 (23.9)	76 (23.9)	1.000
SBP, mmHg	127.3 ± 20.6	124.4 ± 19.2	0.217	129.4 ± 17.3	129.0 ± 22.1	0.611	121.7 ± 17.7	132.1 ± 24.8	<0.001	141.7 ± 23.1	145.2 ± 23.1	0.057
DBP, mmHg	85.5 ± 11.4	78.8 ± 11.9	0.386	77.8 ± 10.0	81.3 ± 14.4	0.128	73.2 ± 11.3	79.2 ± 14.9	0.004	80.4 ± 12.8	82.7 ± 13.8	0.029
FG, mmol/L	5.0 (4.3, 5.6)	5.9 (5.1, 6.9)	0.014	4.7 (4.3, 5.3)	5.7 (4.9, 7.0)	<0.001	5.4 (5.2, 6.0)	6.5 (5.5, 8.5)	<0.001	5.7 (5.3, 6.3)	5.8 (5.3, 6.8)	0.007
TG, mmol/L	1.5 (1.1, 3.0)	1.7 (1.2, 3.3)	0.014	1.1 (0.8, 1.8)	1.5 (1.1, 2.2)	<0.001	1.6 (0.9, 2.1)	1.3 (1.0, 2.0)	0.442	1.2 (0.9, 1.7)	1.4 (1.1, 1.8)	0.218
TCHOL, mmol/L	4.9 ± 1.2	3.9 ± 1.0	0.552	5.1 ± 1.1	4.2 ± 1.0	<0.001	5.3 ± 0.9	5.0 ± 1.3	0.011	4.8 ± 0.9	4.8 ± 0.9	0.919
HDL-C, mmol/L	1.4 (1.2, 1.9)	1.2 (0.8, 1.2)	0.004	1.5 (1.3, 1.8)	1.1 (0.9, 1.3)	<0.001	1.3 (1.2, 1.5)	1.1 (1.0, 1.3)	<0.001	1.4 (1.2, 1.7)	1.3 (1.1, 1.5)	0.006
LDL-C, mmol/L	3.1 ± 0.6	2.1 ± 0.8	0.001	3.5 ± 0.9	2.3 ± 1.0	<0.001	3.6 ± 1.0	3.3 ± 1.0	0.056	2.8 ± 0.8	2.8 ± 0.9	0.730
Hypertension, N (%)	0	9 (75.0)	<0.001	3 (2.8)	66 (61.7)	<0.001	3 (2.9)	42 (40.8)	<0.001	73 (23.0)	174 (54.7)	<0.001
Diabetes, N (%)	0	2 (16.7)	<0.001	2 (1.9)	25 (23.4)	<0.001	1 (1.0)	15 (14.7)	<0.001	26 (8.2)	71 (22.3)	<0.001
Anti-hypertensive medications, N (%)	0	6 (50.0)	<0.001	8 (7.5)	65 (60.7)	0.001	3 (2.9)	20 (19.4)	<0.001	64 (20.1)	150 (47.2)	<0.001
Lipid-lowing medications, N (%)	0	6 (50.0)	<0.001	6 (5.6)	51 (47.7)	<0.001	1 (1.0)	19 (18.4)	<0.001	19 (6.0)	72 (22.6)	<0.001

Continuous variables are presented as mean ± SD or median (25th, 75th), and the distribution differences between cases and controls were tested using ANOVA or Mann-Whitney U test. Categorical variables are presented as N (%), and the proportion differences between cases and controls were tested using Chi-square test. BMI, body mass index; SBP, systolic blood pressure; DBP, diastolic blood pressure; FG, fasting glucose; TCHOL, total serum cholesterol; TG, triglycerides; HDL-C, high-density lipoprotein cholesterol; LDL-C, low-density lipoprotein cholesterol. *Demographic and clinical characteristics recorded at the 2013 baseline of the DFTJ-cohort.

Unexpectedly, we also observed higher LDL levels in controls in comparison with cases in discovery set and validation set 1 (*P* < 0.05), probably due to higher prevalence of statin use in the cases. In the nested case-control study, compared with controls, ACS cases had slightly higher glucose and lower high density lipoprotein levels at baseline (*P* < 0.05).

### Association between plasma cytokines and ACS risk

In the discovery stage, we identified 15 different expression cytokines (all *q*-value < 0.05 and above the detection limit of protein chip) in ACS cases as compared with healthy controls, among which nine cytokines were up-regulated and six cytokines were down-regulated, with MSP as the most regulated cytokine (fold change = 4.65; Table S1 and Fig. S1). Five cytokines, namely osteopontin, CCL23, MSP, BDNF and CRP, met the selection criteria listed in the Material and Method section were selected for next-stage validation (Fig. S1).

In the validation stage, we pooled results from the two case-control studies as similar associations were observed between selected cytokines and ACS in both studies (Table 2). After adjustment for potential covariates, the pooled ORs (95% CI) of ACS for each SD increase of log-transformed osteopontin, CCL23, BDNF, CRP and MSP were 4.64 (3.16–6.79), 3.60 (2.45–5.28), 0.66 (0.49–0.90), 2.67 (1.88–3.79), and 1.33 (0.92–1.91), respectively.

**Table 2:** Adjusted Odds Ratio for Risk of ACS According to Five Selected Plasma Cytokines in Two Case-control Validation Sets.

Cytokines	Validation Set 1		Validation Set 2		Pooled analysis of the two validation sets	
	Adjusted OR (95% CI)	*P*	Adjusted OR (95% CI)	*P*	Adjusted OR (95% CI)	*P*
Osteopontin						
Model 1	4.65 (2.49–8.67)	<0.001	3.86 (2.39–6.22)	<0.001	4.12 (2.81–6.04)	<0.001
Model 2	4.68 (2.48–8.85)	<0.001	3.64 (2.24–5.90)	<0.001	4.04 (2.74–5.95)	<0.001
Model 3	3.89 (1.56–5.47)	0.023	5.98 (3.43–10.44)	<0.001	4.64 (3.16–6.79)	<0.001
CCL23						
Model 1	2.57 (1.57–4.22)	<0.001	9.64 (4.46–20.83)	<0.001	4.70 (1.38–15.97)	0.013
Model 2	2.54 (1.55–4.17)	<0.001	8.80 (4.12–18.81)	<0.001	4.11 (2.76–6.10)	0.009
Model 3	3.01 (1.51–5.99)	0.002	4.37 (2.56–7.46)	<0.001	3.60 (2.45–5.28)	<0.001
BDNF						
Model 1	0.53 (0.41–0.69)	<0.001	0.55 (0.41–0.74)	<0.001	0.54 (0.44–0.66)	<0.001
Model 2	0.51 (0.39–0.67)	<0.001	0.56 (0.42–0.75)	<0.001	0.54 (0.44–0.66)	<0.001
Model 3	0.66 (0.49–0.90)	0.009	0.60 (0.39–0.91)	0.015	0.66 (0.49–0.90)	<0.001
MSP						
Model 1	1.58 (1.08–2.33)	0.019	1.17 (0.82–1.52)	0.484	1.28 (1.01–1.61)	0.039
Model 2	1.54 (1.04–2.27)	0.031	1.18 (0.86–1.62)	0.315	1.29 (1.02–1.63)	0.034
Model 3	2.02 (0.81–5.06)	0.132	1.20 (0.76–1.89)	0.434	1.33 (0.92–1.91)	0.130
CRP						
Model 1	2.56 (1.95–3.38)	<0.001	5.08 (3.27–7.89)	<0.001	3.29 (2.60–4.17)	<0.001
Model 2	2.56 (1.94–3.39)	<0.001	5.17 (3.28–8.14)	<0.001	3.29 (2.60–4.18)	<0.001
Model 3	2.54 (1.39–4.66)	0.003	3.08 (1.87–5.09)	<0.001	2.67 (1.88–3.79)	<0.001

Plasma cytokine levels were ln-transformed prior to analysis, and the ORs were shown as per SD increase of the ln-transformed value of each cytokines. Model 1: Adjusted for age (continuous). Model 2: Additionally, adjusted for BMI (continuous) and smoking status (current, former and never). Model 3: Additionally adjusted for total cholesterol (continuous), low-density lipoprotein cholesterol (continuous), triglycerides (continuous), fasting glucose (continuous), estimated glomerular filtration rate (continuous), systolic blood pressure (continuous), systolic blood pressure (continuous), anti-hypertensive medication (binary), and lipid-lowing medication (binary).

We further investigated whether osteopontin, CCL23, BDNF and CRP were associated with incident ACS in a nested case-control study after a median follow- up of 1.6 years. With adjustment for potential confounding, we observed positive associations of osteopontin and CRP with incident ACS (OR 1.29; 95% CI 1.06–1.57 for osteopontin; OR 1.30; 95% CI 1.02–1.66 for CRP; Table 3). Osteopontin showed a moderate positive correlation with CRP in health controls across all datasets (all *P* < 0.05; Fig. S2). Therefore, we conducted a sensitivity analysis to include both osteopontin and CRP in a single regression model, and the results did not materially change (OR 1.35; 95% CI 1.02–1.78 for osteopontin; OR 1.42; 95% CI 1.06–1.91 for CRP).

**Table 3:** Adjusted Odds Ratio for Risk of ACS According to Four Replicated Plasma Cytokines in Nested Case-control Study.

	Tertiles of cytokines			*P* trends^*^	OR (95% CI) per one SD^**^
	T1	T2	T3		
Osteopontin					
N (cases/controls)	92/120	108/104	118/94		
Median level (ng/ml)	31.68	52.13	82.62		
Model 1	[Ref]	1.36 (0.92–2.00)	1.53 (1.03–2.28)	0.027	1.27 (1.04–1.53)
Model 2	[Ref]	1.37 (0.93–2.01)	1.53 (1.03–2.28)	0.031	1.26 (1.04–1.53)
Model 3	[Ref]	1.55 (1.00–2.41)	1.75 (1.13–2.71)	0.016	1.29 (1.06–1.57)
CCL23					
N (cases/controls)	102/110	113/99	103/109		
Median level (ng/ml)	1.86	2.27	2.91		
Model 1	[Ref]	1.20 (0.71–2.03)	1.08 (0.62–1.86)	0.875	1.02 (0.80–1.29)
Model 2	[Ref]	1.22 (0.72–2.07)	1.08 (0.63–1.87)	0.957	1.02 (0.80–1.29)
Model 3	[Ref]	1.08 (0.64–1.83)	1.00 (0.58–1.73)	0.993	1.03 (0.81–1.34)
BDNF					
N (cases/controls)	110/102	88/124	120/92		
Median level (ng/ml)	10.8	19.91	38.3		
Model 1	[Ref]	1.37 (0.81–2.31)	0.73 (0.43–1.26)	0.334	0.86 (0.68–1.10)
Model 2	[Ref]	1.37 (0.81–2.31)	0.73 (0.42–1.26)	0.337	0.86 (0.68–1.10)
Model 3	[Ref]	1.63 (0.96–2.76)	0.91 (0.52–1.60)	0.791	0.84 (0.65–1.09)
CRP					
N (cases/controls)	95/117	99/113	124/88		
Median level (mg/l)	0.61	1.83	6.28		
Model 1	[Ref]	1.10 (0.75–1.63)	1.49 (1.01–2.21)	0.014	1.45 (1.09–1.91)
Model 2	[Ref]	1.12 (0.76–1.66)	1.53 (1.02–2.32)	0.048	1.48 (1.10–1.98)
Model 3	[Ref]	1.04 (0.67–1.61)	1.50 (0.97–2.33)	0.035	1.30 (1.02–1.66)

Plasma cytokine levels were ln-transformed prior to analysis. Model 1: Adjusted for age (continuous). Model 2: Additionally, adjusted for BMI (continuous) and smoking status (current, former and never). Model 3: Additionally, adjusted for total cholesterol (continuous), low-density lipoprotein cholesterol (continuous), triglycerides (continuous), fasting glucose (continuous), estimated glomerular filtration rate (continuous), systolic blood pressure (continuous), anti-hypertensive medication (binary), and lipid-lowing medication (binary). *P values when we assigned the median value to each group and used this as a continuous variable in linear regression models. **OR for each SD change.

After stratification for onset time windows, we observed a lower association for osteopontin in ≥1 year time period compared with <0.5 year time period and 0.5–1 year time period (*P* > 0.05; Table S3). In stratification analysis, we did not observe evidence for effect modification by baseline covariates (all *P* > 0.05 for interaction; Table S4).

We observed elevated levels of osteopontin and CRP in 82 ACS patients with different ACS subtypes, stenotic vessels and onset time windows after ACS onset (Table S5). Association of osteopontin and CRP with severity and onset time window of ACS.

**Figure 1:**
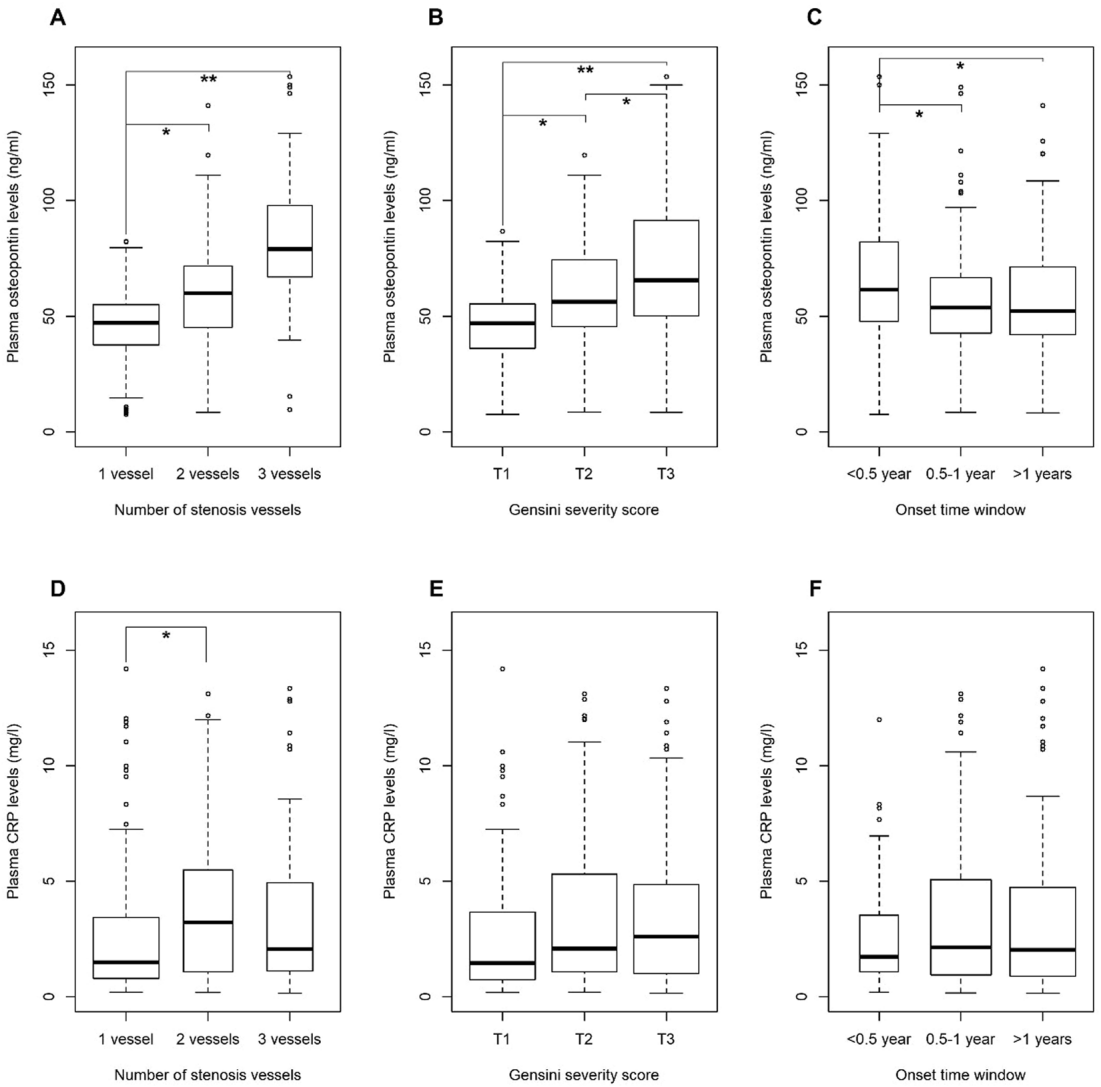
Association of Plasma Osteopontin and CRP Levels with ACS Severity and Onset Time Windows among ACS Cases in the Nested Case-control Study. Higher levels of circulating osteopontin were correlated with higher severity of ACS (**A** and **B**), and earlier ACS onset time (from the time of measurement to the ACS onset; **C**). *represents *P* < 0.05 and **represents *P* < 0.001.

In the nested case-control study, we further identified the association of osteopontin with severity and onset time window of ACS. Among ACS cases, higher plasma osteopontin levels were observed with increasing number of stenotic vessels: the median (25^th^–75^th^ percentile) levels of plasma osteopontin were 47.12 (37.62–55.09) ng/ml in 160 patients with 1-vessel disease, 59.66 (43.78–71.76) ng/ml in 85 patients with 2-vessels disease, and 79.08 (69.51–98.99) ng/ml in 73 patients with 3-vessels disease, respectively (*P* < 0.001, Fig. 1A). There was a positive correlation between plasma osteopontin and Gensini score among ACS patients (r = 0.42; *P* < 0.001; Fig. S3). We also observed a stepwise increase in plasma osteopontin levels with increasing tertile of Gensini score: the median (25^th^–75^th^ percentile) levels of plasma osteopontin were 46.97 (36.06–55.45), 56.16 (45.57–75.73) and 65.43 (50.13–91.83) ng/ml, given Gensini scores of ≤11, 12–31, and >31, respectively; Fig. 1B). Similar trends were observed in ACS patients with shorter onset time windows such that plasma osteopontin were 61.42 (46.83–81.74), 54.27 (43.65–67.52) and 51.87 (41.16–65.97) ng/ml in ACS patients who had their blood collection <0.5 year (49 patients), 0.5–1 years (124 patients), and ≥1 years (145 patients) before ACS onset, respectively (Fig. 1C). Moreover, higher osteopontin levels were observed in patients with ST-segment elevation myocardial infarction (n = 11) compared with patients with UAP (n = 291) (*P* < 0.05; Fig. S4). However, no similar trends were observed for CRP (Figs 1D–F and S4).

### Risk discrimination and reclassification

**Figure 2:**
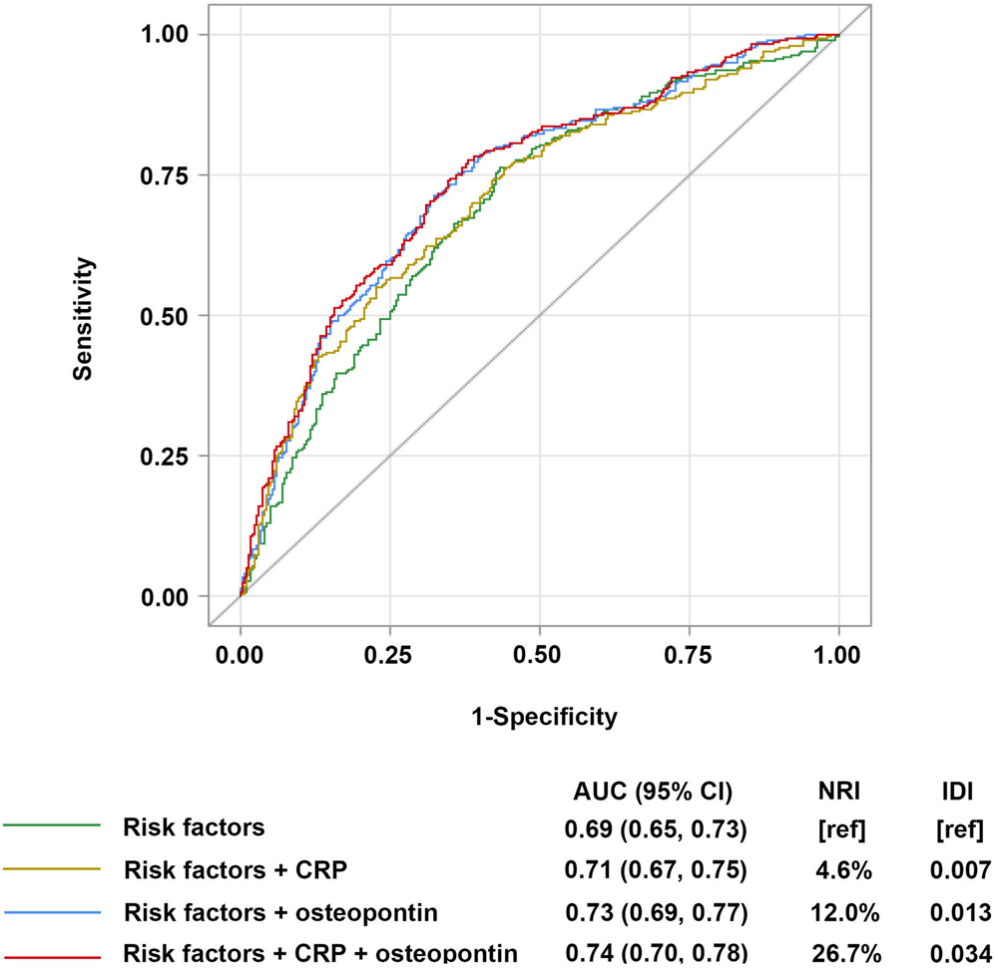
ROC Curve, NRI, IDI Evaluating ACS Cases and Controls in the Nested Case-control Study. The area under the curve (AUC) is shown with its 95% CIs. Traditional risk factors including age, sex, BMI, smoking status, total cholesterol, high-density lipoprotein cholesterol, systolic pressure, status of diabetes and hypertension.

Figure 2 summarized the results of C-index, NRI, and IDI analysis in the nested case-control study. The C-index for incident ACS at 2 years of follow-up was increased from 0.69 to 0.73 and to 0.71, respectively, with the addition of osteopontin and CRP. Moreover, the continuous NRI and IDI metrics were also modestly improved with the addition of osteopontin and CRP separately. However, the largest reclassification was observed for the combination of osteopontin and CRP into the model with an improved area under the curve from 0.69 to 0.74, an improved NRI of 26.7%, and an IDI of 0.034 (all *P* < 0.05).

## Discussion

Through a multi-stage association study, we first identified the prospective associations of osteopontin and CRP with incident ACS. To our best knowledge, this is the first multi-stage study to identify osteopontin as a biomarker for risk and severity of ACS.

In the case-control studies, we identified BDNF and CCL23 in association with ACS. Consistent with our findings, Kaess *et al*.^8^ found an inverse association of BDNF and CVD, supporting the potential role of BDNF in CVD pathogenesis. CCL23 was also reported to participate in inflammatory responses and tube formation, both of which play critical roles in the progression of ACS^9,10^. Our failure to replicate these findings may be due to the elderly population and, relatively modest sample size of the validation study or changes of cytokines levels during disease progression. In addition, we further confirmed the association of higher ACS risk with elevated levels of CRP. In line with our results, Kaptoge *et al*.^6^ found a 37% higher CHD risk per 1-SD elevated log-transferred CRP levels in an updated meta-analysis. In another study from Kaptoge *et al*.^11^, the addition of CRP to the Framingham risk score only increased the C-index by 0.0039, and yielded a NRI of 1.52%, which is similar with our finding that CRP might be a biomarker and only added limited predictive value beyond established risk factors for ACS.

Osteopontin is a multifunctional protein which was thought to play a critical role in atherosclerosis, and there is convincing evidence linking osteopontin to the onset of ACS. Osteopontin is abundantly present in atherosclerotic plaques^12^, it was also reported to interact with integrins^13^ to participate in numerous physiological and pathological events including macrophage chemotaxis, inflammation, and cell survival^14^. Previous studies have found that osteopontin could increase endothelial cell migration via αvβ3 ligand, thus increasing the risk of atherosclerosis^15^. Additionally, osteopontin was considered to be a macrophage-chemotactic stimulant and participated in the recruitment of monocytes-macrophages^16,17^. The potential role of osteopontin in promoting retention of macrophages at sites of chronic inflammation^16^ indicated a possible mechanism linking to ACS onset and progression. Besides, osteopontin was found to be associated with accumulation of calcium in tissues of CHD patients and may, therefore, serve as a surrogate biological marker of coronary arteries calcification^18^. Several case-control studies^18–23^ have examined the associations between osteopontin and CHD, however, with inconsistent findings. Ohmori *et al*.^19^ and Abdel-Azeez *et al*.^20^ found the association of osteopontin with risk and severity of coronary artery disease. Similar to our findings, compared with healthy controls, Tousoulis *et al*.^21^ found higher osteopontin levels among patients with 3-vessels CHD. Higher osteopontin levels were also observed among CHD patients complicated with diabetes^22^. In a recent study, Mohamadpour *et al*.^23^ found an association between osteopontin and CHD, but failed to observe differences in osteopontin levels among CHD subgroups with different narrowed vessels. This discrepancy may be attributed to the small, selected groups of CHD patients recruited after disease onset. However, the association we found in the nested case-control study was much weaker in comparison with that in case-control studies, indicating that osteopontin levels rapidly increased after ACS onset. The dynamic changes before and after ACS onset were further confirmed by the measurement of osteopontin levels in the longitudinal study. Despite an independent association, we found in the present study that osteopontin was modestly correlated with CRP levels, suggesting that osteopontin, as an important inflammatory cytokine, may activate the low-intensive inflammation associated with CRP elevation and other conventional risk factors for ACS, such as hypertension and obesity^18,24^. Although our finding does not establish causality, the comprehensive evaluation of the association with ACS could be useful, given the emerging literature on cytokines as potential targets for drug development^25^.

The finding of osteopontin alone or on top of CRP added to the predictive value for ACS is speculative but attractive. Our study suggested that osteopontin might reflect certain stages of ACS, thus being a useful biomarker in clinically discrimination of the high risk population for ACS. Nevertheless, since this was an observation study, we cannot quantify the clinical benefits associated with the improvement in early diagnostic accuracy, intervention study was still warranted to provide this information.

Our study has several strengths. First, we used the protein microarray to measure 280 cytokines simultaneously in the discovery stage while most previous studies examined only a few selected cytokines. This method allowed direct comparison of circulating plasma cytokines levels, which broadened our abilities to screen cytokines as potential biomarkers for ACS. Moreover, the validation studies were conducted in three independent populations, therefore minimizing the chance of false positive findings. Third, we included a nested case-control study to prospective investigate the associations of cytokines with incident ACS. Last, we observed the association of osteopontin with both early risk and severity of ACS. Risk prediction performance measures further confirmed that adding osteopontin in combination with CRP modestly improved the ability to predict ACS risk.

Some limitations of our study merit consideration. First, our nested case-control participants were only followed up for a median of 1.6 years. Despite that, the rapid increase in the osteopontin levels related to the severity and onset time of ACS has potential implications in clinical practice, suggesting that close monitoring of osteopontin levels in high risk individuals may help clinicians make decisions to reduce disease risk and prevent disease onset. Second, the prediction calculation may be overestimated because plasma cytokines were measured in the nested case-control study instead of the entire cohort. Therefore, our findings could only be interpreted as potential biomarkers, large-scale prospective studies are still warranted. Third, evaluation of ACS severity was based on number of stenosis vessels or segments and the degree of luminal narrowing, further studies were expected to validate the association using more precise scores such as SYNTAX score or calcium score. Last, it was difficult to exclude the possibility of subclinical CHD in controls. Nevertheless, we collected detailed information on symptoms, hospital records, clinical examinations, laboratory tests of blood and urine, and electrocardiogram results to minimize undiagnosed CHD in controls.

In summary, we confirmed the association of osteopontin with incident ACS independent of conventional risk factors in four independent studies of Chinese adults. In addition, our data suggested osteopontin could be a potential biomarker for risk and severity of ACS.

## Methods

### Study design and study populations

**Figure 3:**
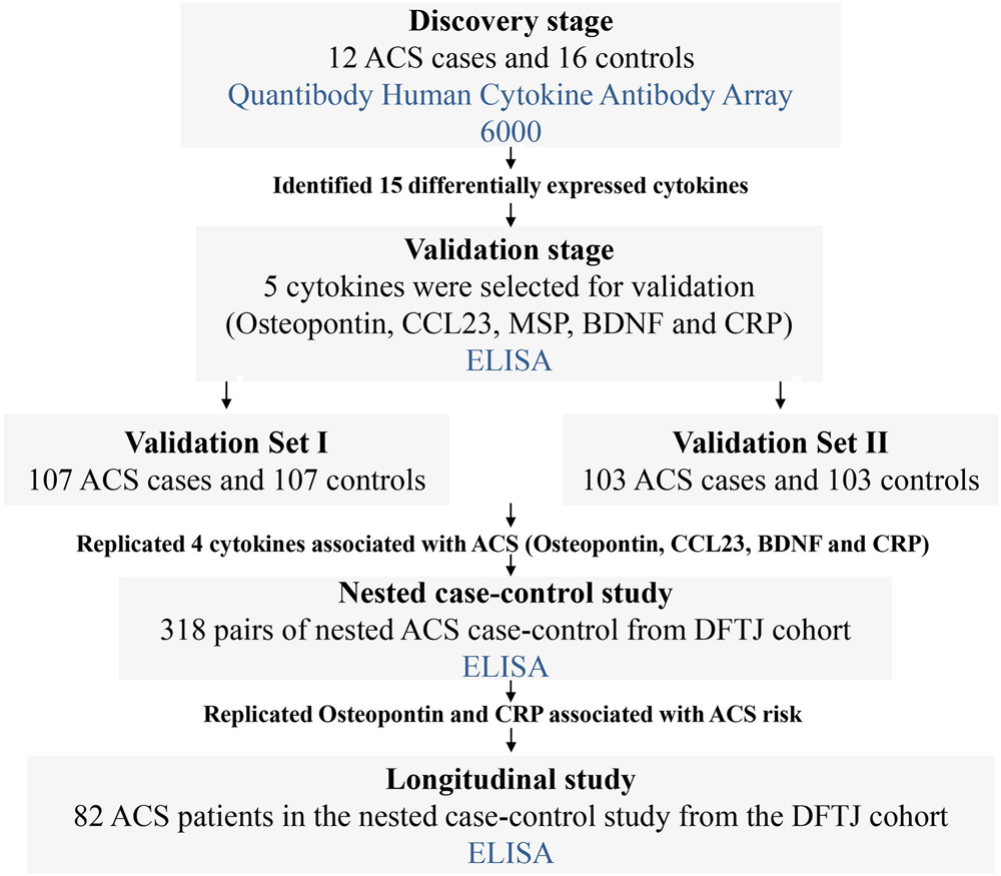
Flowchart of the Study.

We conducted a multi-stage study design in four independent Chinese populations to discover and validate cytokines associated with ACS, including three case-control studies and one prospective nested case-control study (Fig. 3).

In the discovery stage, 12 ACS cases (8 AMIs and 4 UAPs) and 16 frequency matched healthy controls were recruited from Wuhan, Hubei^26^. Clinically confirmed ACS patients, including UAP, NSTEMI, and STEMI, were recruited from Wuhan Union Hospital and Wuhan Central Hospital from 2010 to 2013. ACS were confirmed based on clinical history, symptoms, electrocardiograph, cardiac biomarkers, coronary angiography, risk factors, and/or other clinical examinations according to World Health Organization guidelines^27,28^. Patients who were complicated with congenital heart disease, cardiomyopathy, severe kidney or liver dysfunctions, and cancer were excluded from the study. Healthy controls were randomly selected from Wuhan residents in the Wuhan-Zhuhai cohort during the same period^29^, and were frequency matched on age (±5 years), sex, and BMI (±1 kg/m^2^).

The first validation set recruited 107 ACS cases (76 AMIs and 31 UAPs) from the same sources as in the discovery set. The second validation set recruited 103 ACS cases (76 AMIs and 27 UAPs) from two hospitals in Guangdong, China (Shenzhen Bao’an Hospital and People’s Hospital of Zhuhai, south China) from 2010 to 2013. In the validation studies, healthy controls without cardiovascular disease (CVD) and cancer from Wuhan-Zhuhai cohort were random selected, and were 1:1 matched on age (±5 years), sex, and BMI (±1 kg/m^2^). In the nested case-control study, we enrolled 318 ACS (27 AMIs and 291 UAPs) case-control pairs from the Dongfeng-Tongji cohort. Details of the Dongfeng-Tongji cohort have been reported elsewhere^30^. Briefly, between April and November 2013, we conducted questionnaire inquiries (including major chronic diseases) and physical examinations among retired employees from the Dongfeng Motor Corporation. The company has its own affiliated hospitals and comprehensive health care system, which allowed us to track for morbidity and mortality records of all participants. Baseline CVD and cancer cases were excluded based on self-report or medical records. Incident ACS cases were identified through review of medical insurance documents, hospital records, and death certificates during the follow-up until June 2015. All the diagnostic information and medical records for participants with diagnosed ACS were carefully checked and adjudicated by a group of trained physicians who were blinded to cytokine data. After a median of 1.6 years of follow-up, 318 eligible incident ACS cases were included in the present analysis. Controls were randomly selected from participants who were free of CVD and cancer at baseline and were also CVD-free up to June 2015, and were 1:1 matched on age (±5 years), sex, BMI (±1 kg/m^2^) and smoking status (current, former and never) to incident ACS cases.

In the longitudinal study, we recruited 82 out of the 318 ACS cases from the nested case-control study who were admitted to the Dongfeng Central Hospital (affiliated with the Dongfeng Motor Corporation) in Shiyan City (central China) from February 2014 to June 2015. Blood samples were drawn within 24 hours of the ACS onset before any medication use. We then compared their blood cytokine levels at baseline (2013) and immediately after ACS onset (2014–2015).

The study was approved by Ethics Committee of the Tongji Medical College and all participating hospitals. All participants signed informed consents and all experiments were performed in accordance with relevant guidelines and regulations.

### Biomarker measurements

For the discovery set, 280 plasma cytokines were quantitated using Quantibody Human Cytokine Antibody Array 6000 (Raybiotech Inc., Georgia, USA) according to the manufacturer’s procedure^31^. Five cytokines (osteopontin, chemokine ligand 23 [CCL23], macrophage stimulating protein [MSP], brain derived neurotrophic factor [BDNF], and CRP) were selected for the next stage validation. Details of the selection criteria were described under statistical analyses. In the validation stage, plasma levels of osteopontin (Cat. # SOST00, R&D, USA), BDNF (Cat. # SBD00, R&D, USA), and CRP (Cat. # SCRP00, R&D, USA), MSP (Cat. # ab100612, Abcam, USA) and CCL23 (Cat. # ab100611, Abcam, USA) were determined by high-sensitivity enzyme linked immunosorbent assay (ELISA) kits.

In the nested case-control study, overnight fasted blood samples were collected during the physical examination at baseline in 2013, before the ACS onset. Plasma biomarkers were measured from plasma samples that had been stored at −80 °C immediately after collection and have not been thawed until analysis. To avoid batch effect, all matched case-control pairs and the measurement sequence were randomized before analysis. Measurements of each case-control pair were performed in duplicate in the same plate. Intra-assay and inter-assay coefficients of variation for all the measurements were <5% and <10%, respectively.

### Statistical analysis

The baseline characteristics of ACS cases and controls were compared using one-way analysis of variance (ANOVA) or Mann-Whitney U test for continuous variables and the chi-square for categorical variables. The cytokine microarray data were analyzed using Significance Analysis of Microarray (SAM) 3.00 algorithm (http://statweb.stanford.edu/~tibs/SAM/index.html). SAM assigns each cytokine a *d*-score based on a multi-comparison analysis of expression changes and indicates significance by fold change and *q*-value (*q*-value was defined as the false discovery rate [FDR] adjusted *p*-value). Five cytokines (osteopontin, CCL23, MSP, BDNF and CRP) were selected for the next stage validation based on the following three selection criteria: 1) at least a 2-fold higher (fold change ≥2) or lower (fold change ≤0.5) expression in the ACS group compared with the control group; 2) *q*-value <0.05 between ACS and control group; 3) above the limit of detection in each individual. Cytokine levels measured by ELISA were natural-logarithm transformed before analysis. Conditional logistic regression analysis was used to calculate the odds ratios (ORs) of cytokines with adjustment for age, sex, BMI, drinking status, smoking status, systolic blood pressure (SBP), total serum cholesterol, low density lipoprotein (LDL), triglyceride, estimated glomerular filtration rate (eGFR; in mL/min per 1.73 m^2^), medication of anti-hypertensive and lipid-lowing medications. Correlation coefficients between validate cytokines and blood lipid levels in controls were estimated by Spearman partial correlation coefficients with adjustment for age, sex, BMI, and smoking status.

In the nested case-control study, cytokines were divided into tertiles, from the lowest to highest levels, on the basis of the distributions among the controls. To test the linear trends of the associations between cytokines and ACS, we used the median levels of cytokines in each tertile as continuous variables. To investigate the association of cytokines with ACS severity, two coronary scoring systems were used to evaluate ACS severity: the most adopted clinical 1- to 3- vessels disease score^32,33^ and the Gensini score^34^. All ACS patients were classified according to the number of >50% stenotic vessels, and a ≥50% narrowing of the left main coronary artery was considered as 2-vessels disease, based on which ACS cases were categorized into 1-vessel disease, 2-vessels disease, or 3-vessels disease^27,28^. Among 291 ACS patients with sufficient information to calculate Gensini score, each segment score equals weighting factor (5 for the left main, 2.5 for the proximal circumflex [Cx] and left anterior descending [LAD], 1.5 for the mid LAD, 1 for the right coronary artery, the obtuse marginal branch of Cx, the distal Cx, the posterior descending artery, and the first diagonal branch and the distal LAD, and 0.5 for the posterolateral system and the second diagonal branch, respectively) multiplied by a severity score that represents the percentage luminal diameter reduction of the coronary artery lumen (32 for 100%, 16 for 99%, 8 for 90%, 4 for 75%, 2 for 50%, and 1 for 25% lumen diameter reduction, respectively). Scoring of all coronary angiograms was done by two investigators who were unware of clinical and laboratory data. Onset time window was defined as the time period from blood sample collection to ACS onset. We stratified ACS nested case-controls into <0.5 year, 0.5–1 year and ≥1 year groups according to onset time window of ACS cases. Stratified analysis was conducted with unconditional logistic regression models to evaluate associations between osteopontin levels and ACS risk in each stratum of traditional cardiovascular risk factors. For the nested case-control analysis, we constructed models by adding osteopontin and CRP independently or simultaneously to the Framingham risk score, and looked for the additive value of cytokines. The discrimination value of cytokines for the ACS prediction was illustrated by comparing area under the ROC curve (AUC)^35^, while the added predictive ability of cytokines combined with Framingham risk score was assessed by the integrated discrimination improvement (IDI) index and net reclassification index (NRI)^36,37^. We conducted all analysis using SAS version 9.3 (SAS institute Inc., Cary, NC) and a two-sided *P*-value < 0.05 was considered statistically significant.

## Supplementary information

Supplementary file_A multi-stage association study of plasma cytokines identifies osteopontin as a biomarker for acute coronary syndrome risk and severity

## Data Availability

The datasets generated and/or analysed during the current study are available from the corresponding author on reasonable request.

## References

[1] Go AS (2014). American Heart Association Statistics Committee and Stroke Statistics Subcommittee; Heart disease and stroke statistics-2014 update: a report from the American Heart Association. Circulation.

[2] O’Connor RE (2010). Part 9: Acute coronary syndromes: 2010 International consensus on cardiopulmonary resuscitation and emergency cardiovascular care science with treatment recommendations. Circulation.

[3] Hansson GK (2005). Inflammation, atherosclerosis, and coronary artery disease. N Engl J Med.

[4] Armstrong EJ, Morrow DA, Sabatine MS (2006). Inflammatory biomarkers in acute coronary syndromes: part III: biomarkers of oxidative stress and angiogenic growth factors. Circulation.

[5] Zhang JM, An J (2007). Cytokines, inflammation, and pain. Int Anesthesiol Clin.

[6] Kaptoge S (2010). C-reactive protein concentration and risk of coronary heart disease, stroke, and mortality: an individual participant meta-analysis. Lancet.

[7] Kaptoge S (2014). Inflammatory cytokines and risk of coronary heart disease: new prospective study and updated meta-analysis. Eur Heart J.

[8] Kaess BM (2015). Circulating brain-derived neurotrophic factor concentrations and the risk of cardiovascular disease in the community. J Am Heart Assoc.

[9] Kim J, Kim YS, Ko J (2010). CK beta 8/CCL23 induces cell migration via the Gi/Go protein/PLC/PKC delta/NF-kappa B and is involved in inflammatory responses. Life Sci.

[10] Son KN, Hwang J, Kwon BS, Kim J (2006). Human CC chemokine CCL23 enhances expression of matrix metalloproteinase-2 and invasion of vascular endothelial cells. Biochem Biophys Res Commun.

[11] The emerging risk factors collaboration (2012). C-reactive protein, fibrinogen, and cardiovascular disease prediction. N Engl J Med.

[12] Ikeda T, Shirasawa T, Esaki Y, Yoshiki S, Hirokawa K (1993). Osteopontin mRNA is expressed by smooth muscle-derived foam cells in human atherosclerotic lesions of the aorta. J Clin Invest.

[13] Lund SA (2013). Osteopontin mediates macrophage chemotaxis via α4 and α9 integrins and survival via the α4 integrin. J Cell Biochem.

[14] Speer MY (2005). Smooth muscle cells deficient in osteopontin have enhanced susceptibility to calcification *in vitro*. Cardiovasc Res.

[15] Icer MA, Gezmen-Karadag M (2018). The multiple functions and mechanisms of osteopontin. Clin Biochem.

[16] Scatena M, Liaw L, Giachelli CM (2007). Osteopontin: a multifunctional molecule regulating chronic inflammation and vascular disease. Arterioscler Thromb vasc Biol.

[17] Mazzali M (2002). Osteopontin—a molecule for all seasons. QJM- Int J Med.

[18] de Castro Brás LE (2015). Osteopontin: A major player on hypertension-induced vascular remodeling. J Mol Cell Cardiol.

[19] Ohmori R (2003). Plasma osteopontin levels are associated with the presence and extent of coronary artery disease. Atherosclerosis.

[20] Abdel-Azeez HA, Al-Zaky M (2010). Plasma osteopontin as a predictor of coronary artery disease: association with echocardiographic characteristics of atherosclerosis. J Clin Lab Anal.

[21] Tousoulis D (2013). Serum osteoprotegerin and osteopontin levels are associated with arterial stiffness and the presence and severity of coronary artery disease. Int J Cardiol.

[22] Yan X (2010). Plasma concentrations of osteopontin, but not thrombin-cleaved osteopontin, are associated with the presence and severity of nephropathy and coronary artery disease in patients with type 2 diabetes mellitus. Cardiovasc Diabetol.

[23] Mohamadpour AH (2015). Serum osteopontin concentrations in relation to coronary artery disease. Arch Med Res.

[24] Gomez-Ambrosi J (2007). Plasma osteopontin levels and expression in adipose tissue are increased in obesity. J Clin Endocrinol Metab.

[25] Tousoulis D, Oikonomou E, Economou EK, Crea F, Kaski JC (2016). Inflammatory cytokines in atherosclerosis: current therapeutic approaches. Eur Heart J.

[26] Li J (2017). Genome-Wide Analysis of DNA Methylation and Acute Coronary Syndrome. Circ Res.

[27] Wright RS (2011). 2011 ACCF/AHA focused update incorporated into the ACC/AHA 2007 Guidelines for the management of patients with unstable angina/non-ST-elevation myocardial infarction: a report of the American college of cardiology foundation/American heart association task force on practice guidelines developed in collaboration with the American academy of family physicians, society for cardiovascular angiography and interventions, and the society of thoracic surgeons. J Am Coll Cardiol.

[28] Bassand JP (2008). Guidelines for the diagnosis and treatment of non-ST-segment elevation acute coronary syndromes. Rev Port Cardiol.

[29] Song Y (2014). The Wuhan-Zhuhai (WHZH) cohort study of environmental air particulate matter and the pathogenesis of cardiopulmonary diseases: study design, methods and baseline characteristics of the cohort. BMC Public Health.

[30] Wang F (2013). Cohort Profile: the Dongfeng-Tongji cohort study of retired workers. Int J Epidemiol.

[31] Yang B (2016). Exposure to polycyclic aromatic hydrocarbons, plasma cytokines, and heart rate variability. Sci Rep.

[32] Guidelines for coronary angiography (1987). A report of the American College of Cardiology/American Heart Association Task Force on Assessment of Diagnostic and Therapeutic Cardiovascular Procedures (Subcommittee on Coronary Angiography). J Am Coll Cardiol.

[33] Spears JR (1983). Computerized image analysis for quantitative measurement of vessel diameter from cineangiograms. Circulation.

[34] Gensini GG (1983). A more meaningful scoring system for determining the severity of coronary heart disease. Am J Cardiol.

[35] Heagerty PJ, Zheng Y (2005). Survival model predictive accuracy and ROC curves. Biometrics.

[36] Pencina MJ, D’Agostino RB, Steyerberg EW (2011). Extensions of net reclassification improvement calculations to measure usefulness of new biomarkers. Stat Med.

[37] Sundström J, Byberg L, Gedeborg R, Michaëlsson K, Berglund L (2011). Useful tests of usefulness of new risk factors: tools for assessing reclassification and discrimination. Scand J Public Health.

