# Supplementary material for: A multi-stage association study of plasma cytokines identifies osteopontin as a biomarker for acute coronary syndrome risk and severity
Source: Sci Rep. 2019 Mar 26;9:5121. doi: 10.1038/s41598-019-41577-4 (PMC6435654; doi:10.1038/s41598-019-41577-4)
Supplement: Supplementary file 1 — Supplementary file_A multi-stage association study of plasma cytokines identifies osteopontin as a biomarker for acute coronary syndrome risk and severity [file 41598_2019_41577_MOESM1_ESM.docx]

**Supplementary file**

**A multi-stage association study of plasma cytokines identifies osteopontin as a biomarker for acute coronary syndrome risk and severity**

Kuai Yu^1†^, MD; Binyao Yang^1,8†^, PhD; Haijing Jiang^1^, MD; Jun Li^1^, PhD; Kai Yan^1^, MD; Xuezhen Liu^1^, MD; Lue Zhou^1^, MD; Handong Yang^2^, MD; Xiulou Li^2^, MD; Xinwen Min^2^, MD; Ce Zhang^2^, MD; Xiaoting Luo^3^, MD; Wenhua Mei^3^, MD; Shunchang Sun^4^, MD; Liyun Zhang^5^, MD; Xiang Cheng^6^, MD, PhD; Meian He^1^, MD, PhD; Xiaomin Zhang^1^, MD, PhD; An Pan^1^, PhD; Frank B. Hu^7^, MD, PhD; Tangchun Wu^1^, MD, PhD

^†^Equal contributions

| **Table of Contents Page** | | |
| --- | --- | --- |
|  | | **page** |
| **Figure S1** | Heatmap of the 15 differentially expressed (*q*-value<0.05) cytokines in ACS cases and controls in the discovery stage. | 3 |
| **Figure S2** | Adjusted spearman partial-correlation coefficients between replicated cytokines and blood lipid levels among four different study sets. | 4 |
| **Figure S3** | The correlation of plasma osteopontin levels and Gensini severity score among ACS patients in the nested case-control study. | 5 |
| **Figure S4** | Plasma osteopontin and CRP levels in different subtypes of ACS in the nested case-control study. | 6 |
| **Table S1** | Plasma cytokines measured with cytokine antibody array (Quantibody® Human Cytokine Antibody Array 6000) in the discovery stage. | 7-20 |
| **Table S2** | Levels of cytokines in each of the validation populations. | 21 |
| **Table S3** | Association between replicated cytokines (osteopontin and CRP) and incident ACS in different onset time groups in nested case-control study. | 22 |
| **Table S4** | Association of incident ACS with replicated cytokines stratified by established risk factors in the nested case-control study. | 23 |
| **Table S5** | Plasma osteopontin and CRP levels in different subtypes, onset time groups and stenotic vessels before and after ACS onset. | 24 |

**Supplementary Figure 1. Heatmap of the 15 differentially expressed (*q*-value<0.05) cytokines in ACS cases and controls in the discovery stage.** The rows of the microarray heatmap represent cytokines, and the columns represent samples. Each cell is colorized based on the expression level of that cytokine in that sample. The color gradient for each sample ranges from highest (red) to lowest (blue). Clusters were obtained by combining the average intensity values of all ACS cases and controls.


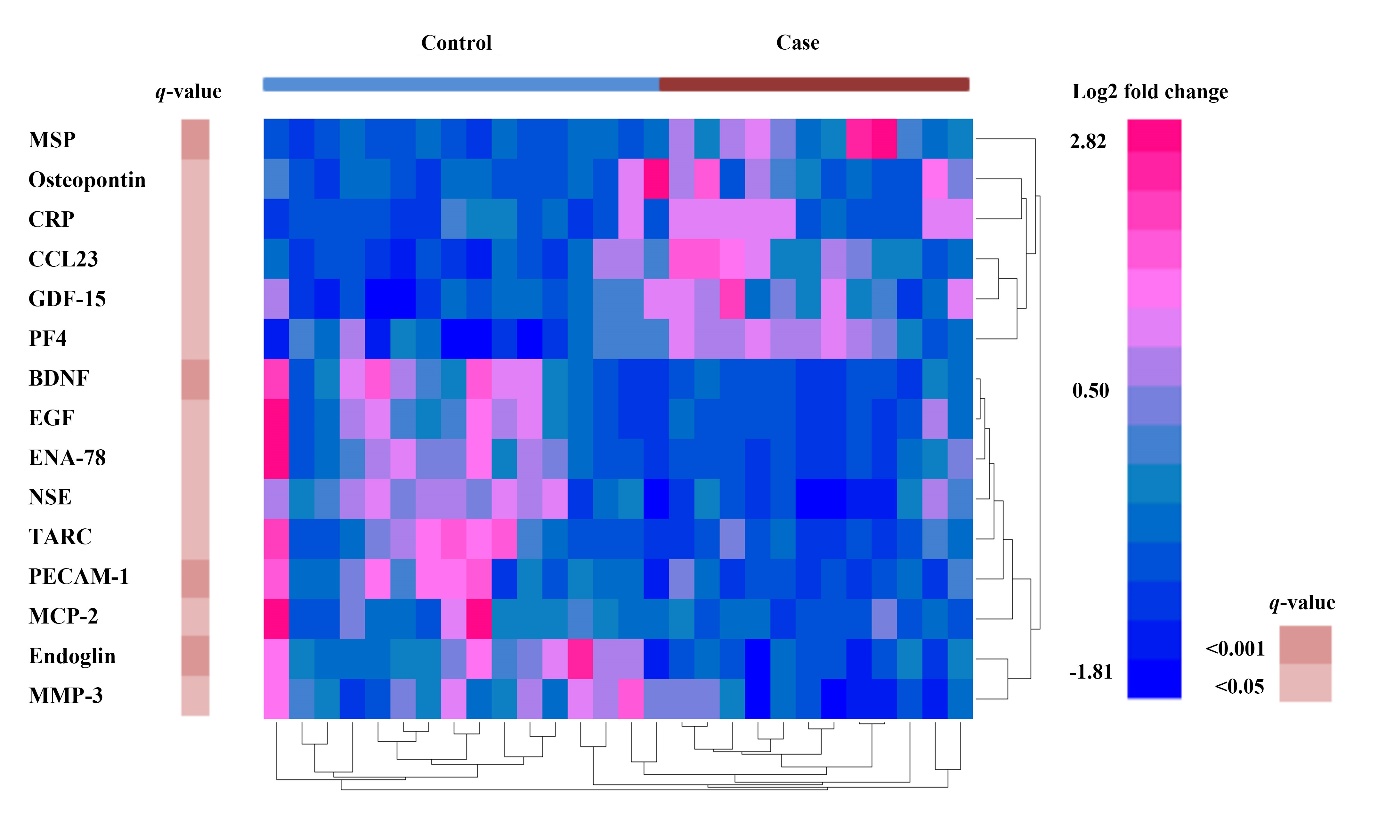


**Supplementary Figure 2. Adjusted spearman partial-correlation coefficients between replicated cytokines and blood lipid levels in controls among four different study sets. ***


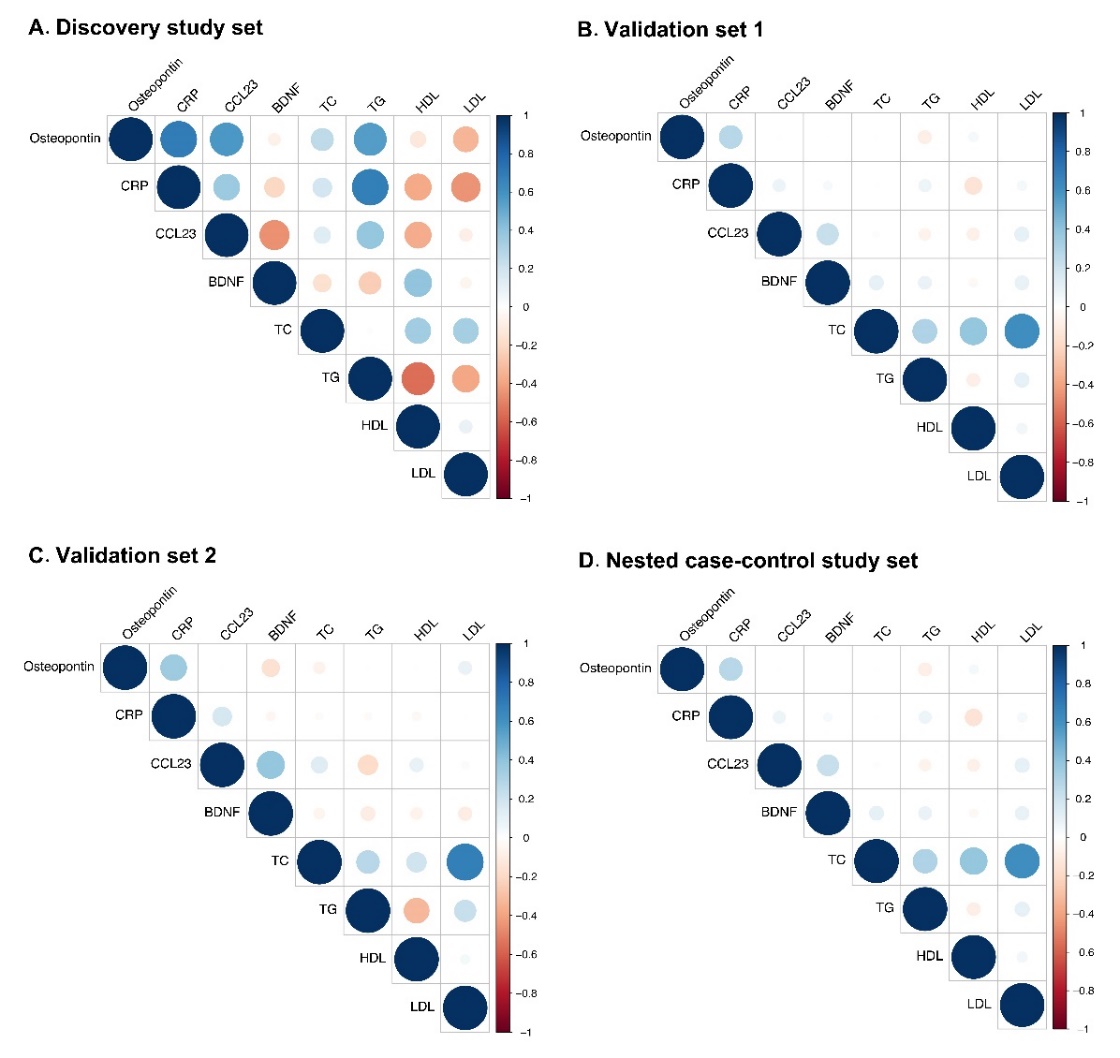


* Spearman partial correlation with adjustment for each other as well as age, sex, BMI and smoking status.

**Supplementary Figure 3. The correlation of plasma osteopontin levels and Gensini severity score among ACS patients in the nested case-control study.**

**
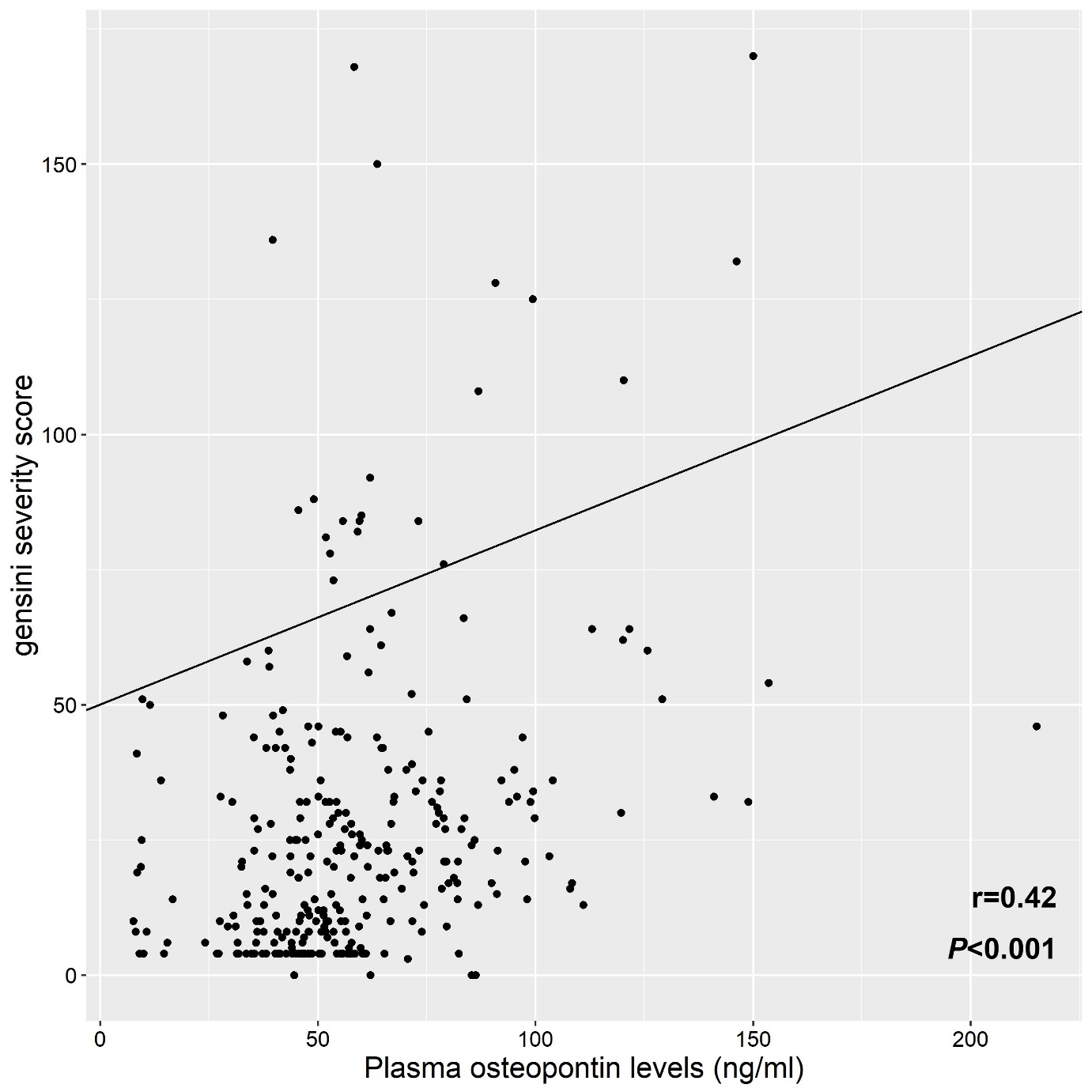
**

**Supplementary Figure 4. Plasma osteopontin and CRP levels in different subtypes of ACS in the nested case-control study.** Higher circulating osteopontin levels were observed in STEMI compared with those in UAP.

**
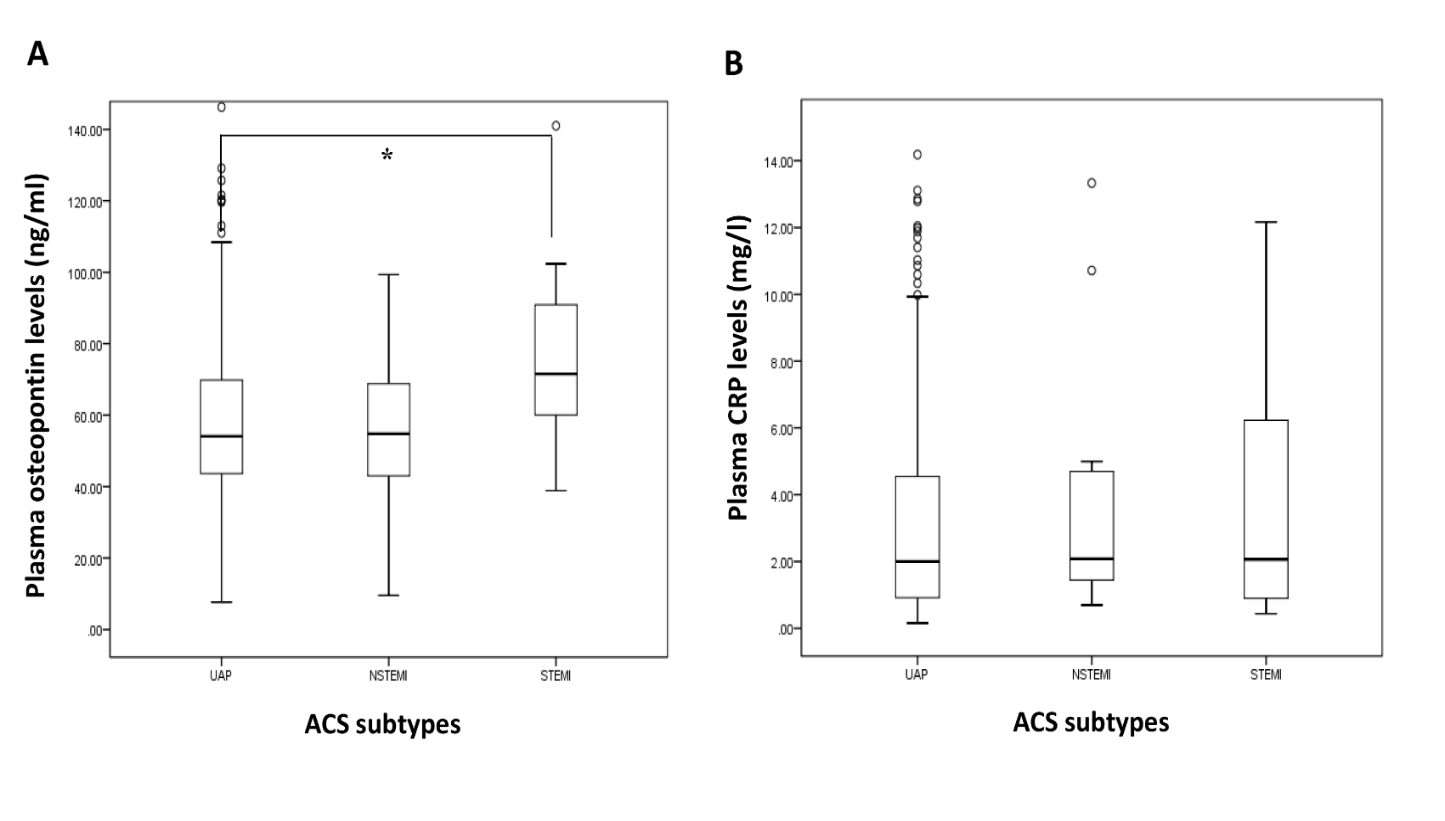
**

* represents *P*<0.05.

**Supplementary Table 1. Plasma cytokines measured with cytokine antibody array (Quantibody® Human Cytokine Antibody Array 6000) in the discovery stage.**

| **NO** | **Cytokines** | **Official symbol** | **Full name** | **Score(d)** | **Fold Change*** | ***Q*-value (%)** |
| --- | --- | --- | --- | --- | --- | --- |
| 1 | Adiponectin | ADIPOQ | Adiponectin, C1Q and collagen domain containing | 0.4516 | 1.0484 | 30.2559 |
| 2 | Adipsin | Adipsin | Adipsin | 1.0305 | 1.1209 | 27.3549 |
| 3 | Angiogenin | ANG | Angiogenin, ribonuclease, rnase A family, 5 | 0.3215 | 1.0266 | 30.2559 |
| 4 | ANG-2 | ANGPT2 | Angiopoietin 2 | 0.8752 | 1.2141 | 27.9221 |
| 5 | APRIL | TNFSF13 | Tumor necrosis factor (ligand) superfamily, member 13 | 1.0187 | 1.1952 | 27.3549 |
| 6 | AR | AR | Androgen receptor | 0.8396 | 1.0729 | 27.9221 |
| 7 | B2M | B2M | Beta-2-microglobulin | 0.8835 | 1.9015 | 27.9221 |
| 8 | B7-1/CD80 | CD80 | CD80 molecule | 0.2734 | 1.7966 | 30.2559 |
| 9 | BCAM | BCAM | Basal cell adhesion molecule (Lutheran blood group) | 1.1223 | 2.9537 | 27.3549 |
| 10 | bFGF | FGF2 | Fibroblast growth factor 2 (basic) | 0.4175 | 1.0239 | 30.2559 |
| 11 | BLC | CXCL13 | Chemokine (C-X-C motif) ligand 13 | 0.8354 | 1.6568 | 27.9221 |
| 12 | BMP-4 | BMP4 | Bone morphogenetic protein 4 | 0.5753 | 0.7958 | 30.2559 |
| 13 | BMP-5 | BMP5 | Bone morphogenetic protein 5 | 0.6817 | 0.2805 | 30.2559 |
| 14 | BMP-7 | BMP 7 | Bone morphogenetic protein 7 | 0.8888 | 0.9094 | 27.9221 |
| 15 | CA125 | MUC16 | Mucin 16, cell surface associated | 1.2656 | 5.0175 | 23.3005 |
| 16 | CA15-3 | MUC1 | Mucin 1, cell surface associated | 1.0452 | 10.3524 | 27.3549 |
| 17 | CD200 | CD200 | CD200 molecule | 0.3627 | 1.0715 | 30.2559 |
| 18 | CD97 | CD97 | CD97 molecule | 0.8678 | 0.9286 | 27.9221 |
| 19 | CEACAM-1 | CEACAM1 | Carcinoembryonic antigen-related cell adhesion molecule 1 | 0.2488 | 1.0820 | 30.2559 |
| 20 | CRP | CRP | C-reactive protein, pentraxin-related | 2.8721 | 3.0101 | 4.3261 |
| 21 | CTACK | CCL27 | Chemokine (C-C motif) ligand 27 | 2.1550 | 1.5270 | 20.8625 |
| 22 | DAN/NBL1 | NBL1 | Neuroblastoma 1, DAN family BMP antagonist | 0.5731 | 0.9473 | 30.2559 |
| 23 | Eotaxin/CCL11 | CCL11 | Chemokine (C-C motif) ligand 11 | 0.0549 | 0.9879 | 30.2559 |
| 24 | Eotaxin-3/CCL26 | CCL26 | Chemokine (C-C motif) ligand 26 | 1.0783 | 1.0990 | 27.3549 |
| 25 | ErbB2 | ERBB2 | V-erb-b2 avian erythroblastic leukemia viral oncogene homolog 2 | 0.0671 | 0.4035 | 30.2559 |
| 26 | Fas | FAS | Fas cell surface death receptor | 0.3769 | 0.3907 | 30.2559 |
| 27 | Ferritin | FTL | Ferritin, light polypeptide | 2.0056 | 1.5950 | 20.8625 |
| 28 | FGF-7 | FGF7 | Fibroblast growth factor 7 | 1.3055 | 0.9998 | 23.3005 |
| 29 | Galectin-3 | LGALS3 | Lectin, galactoside-binding, soluble, 3 | 0.0723 | 0.9723 | 30.2559 |
| 30 | GDF-15 | GDF15 | Growth differentiation factor 15 | 3.0114 | 1.6129 | 2.9252 |
| 31 | GDNF | GDNF | Glial cell derived neurotrophic factor | 0.7775 | 0.9155 | 30.2559 |
| 32 | GH | GH1 | Growth hormone 1 | 1.8662 | 2.4117 | 21.5539 |
| 33 | GITR/TNFRSF18 | TNFRSF18 | Tumor necrosis factor receptor superfamily, member 18 | 1.0211 | 0.8097 | 27.3549 |
| 34 | HB-EGF | HBEGF | Heparin-binding EGF-like growth factor | 0.2454 | 1.3774 | 30.2559 |
| 35 | HCC-1 | CCL14 | Chemokine (C-C motif) ligand 14 | 1.4614 | 1.1913 | 23.3005 |
| 36 | I-309/CCL1 | CCL1 | Chemokine (C-C motif) ligand 1 | 1.3200 | 1.7318 | 23.3005 |
| 37 | ICAM-1 | ICAM1 | Intercellular adhesion molecule 1 | 1.1301 | 1.1839 | 27.3549 |
| 38 | IGFBP-3 | IGFBP3 | Insulin-like growth factor binding protein 3 | 0.7430 | 1.1539 | 30.2559 |
| 39 | IGFBP-4 | IGFBP4 | Insulin-like growth factor binding protein 4 | 2.1799 | 1.3985 | 20.8625 |
| 40 | IGF-II | IGF2 | Insulin-like growth factor 2 (somatomedin A) | 0.9138 | 18.3745 | 27.9221 |
| 41 | IL-1 R4/ST2 | ST2 | Suppression of tumorigenicity 2 | 1.0810 | 1.5144 | 27.3549 |
| 42 | IL-1 RI | IL1R1 | Interleukin-1 receptor type I | 0.4788 | 1.0922 | 30.2559 |
| 43 | IL-10 | IL10 | Interleukin 10 | 0.1644 | 0.3694 | 30.2559 |
| 44 | IL-10 Rβ | IL10RB | Interleukin 10 receptor, beta | 0.8762 | 1.2225 | 27.9221 |
| 45 | IL-12p40 | IL12B | Interleukin 12B (natural killer cell stimulatory factor 2, cytotoxic lymphocyte maturation factor 2, p40) | 1.6128 | 1.8210 | 23.3005 |
| 46 | IL-1β | IL1B | Interleukin 1, beta | 1.2261 | 3.5740 | 27.3549 |
| 47 | IL-2Rγ | IL2RG | Interleukin 2 receptor, gamma | 1.3429 | 1.5406 | 23.3005 |
| 48 | IL-21R | IL21R | Interleukin 21 receptor | 0.1967 | 2.3082 | 30.2559 |
| 49 | IL-28A | IFNL2 | Interferon, lambda 2 | 2.6407 | 3.4761 | 6.3416 |
| 50 | IL-6 sR | IL6R | Interleukin 6 receptor | 0.4269 | 1.0406 | 30.2559 |
| 51 | Insulin | INS | Insulin | 0.5555 | 1.0348 | 30.2559 |
| 52 | LGMN | LGMN | Legumain | 1.0905 | 1.1644 | 27.3549 |
| 53 | Lep | LEP | Leptin | 0.6974 | 1.2497 | 30.2559 |
| 54 | LIF | LIF | Leukemia inhibitory factor | 0.9628 | 27.0440 | 27.4066 |
| 55 | LYVE-1 | LYVE1 | Lymphatic vessel endothelial hyaluronic acid receptor 1 | 0.3510 | 1.0273 | 30.2559 |
| 56 | MBL | MBL2 | Mannose-binding lectin (protein C) 2, soluble | 0.5353 | 1.0464 | 30.2559 |
| 57 | MCP-1 | CCL2 | Chemokine (C-C motif) ligand 2 | 1.3635 | 1.4905 | 23.3005 |
| 58 | MCP-4 | CCL13 | Chemokine (C-C motif) ligand 13 | 0.6005 | 0.8791 | 30.2559 |
| 59 | MCSF | CSF1 | Colony stimulating factor 1 (macrophage) | 0.6559 | 2.1390 | 30.2559 |
| 60 | MDC | CCL22 | Chemokine (C-C motif) ligand 22 | 1.1515 | 0.7428 | 27.3549 |
| 61 | MICA | MICA | MHC class I polypeptide-related sequence A | 0.6821 | 1.2174 | 30.2559 |
| 62 | MICB | MICB | MHC class I polypeptide-related sequence B | 0.9771 | 0.7728 | 27.4066 |
| 63 | MIF | MIF | Macrophage migration inhibitory factor (glycosylation-inhibiting factor) | 1.0247 | 1.1401 | 27.3549 |
| 64 | MIP-3α/CCL20 | CCL20 | Chemokine (C-C motif) ligand 20 | 0.9066 | 1.7116 | 27.9221 |
| 65 | MIP-3β/CCL19 | CCL19 | Chemokine (C-C motif) ligand 19 | 0.4704 | 0.5484 | 30.2559 |
| 66 | MMP-10 | MMP10 | Matrix metallopeptidase 10 (stromelysin 2) | 0.3655 | 56.4246 | 30.2559 |
| 67 | MMP-13 | MMP13 | Matrix metallopeptidase 13 (collagenase 3) | 0.6042 | 4.5885 | 30.2559 |
| 68 | MMP-2 | MMP2 | Matrix metallopeptidase 2 (gelatinase A, 72kda gelatinase, 72kda type IV collagenase) | 0.2357 | 0.3979 | 30.2559 |
| 69 | MMP-9 | MMP9 | Matrix metallopeptidase 9 (gelatinase B, 92kda gelatinase, 92kda type IV collagenase) | 0.3624 | 1.0440 | 30.2559 |
| 70 | MPIF-1/CCL23 | CCL23 | Chemokine (C-C motif) ligand 23 | 3.0211 | 1.9380 | 2.9252 |
| 71 | MSP | MST1 | Macrophage stimulating 1 (hepatocyte growth factor-like) | 4.6512 | 3.8049 | 0.0000 |
| 72 | Notch-1 | NOTCH1 | Notch 1 | 1.0035 | 1.1402 | 27.3549 |
| 73 | NRG1-β1 | NRG1 | Neuregulin 1 | 2.0458 | 3.1397 | 20.8625 |
| 74 | NT-3 | NTF3 | Neurotrophin 3 | 1.1190 | 1.6709 | 27.3549 |
| 75 | NT-4 | NTF4 | Neurotrophin 4 | 1.0682 | 1.4133 | 27.3549 |
| 76 | Osteopontin/SPP1 | SPP1 | Secreted phosphoprotein 1 | 2.8720 | 2.8434 | 2.9252 |
| 77 | OSM | OSM | Oncostatin M | 0.0000 | 1.0000 | 30.2559 |
| 78 | PARC/CCL18 | CCL18 | Chemokine (C-C motif) ligand 18 (pulmonary and activation-regulated) | 1.9504 | 1.2224 | 21.5539 |
| 79 | PF4 | PF4 | Platelet factor 4 | 2.7756 | 1.5865 | 2.9252 |
| 80 | PGRP-5 | pglyrp5 | Peptidoglycan recognition proteins 5 | 0.5473 | 1.0482 | 30.2559 |
| 81 | PIGF | PIGF | Phosphatidylinositol glycan anchor biosynthesis, class F | 1.5025 | 1.3708 | 23.3005 |
| 82 | Procalcitonin/CALCA | CALCA | Calcitonin-related polypeptide alpha | 1.8066 | 2.0235 | 21.5539 |
| 83 | RAGE/AGER | AGER | Advanced glycosylation end product-specific receptor | 0.1245 | 1.0020 | 30.2559 |
| 84 | RANTES/CCL5 | CCL5 | C-C motif chemokine 5 | 1.2042 | 1.2037 | 27.3549 |
| 85 | SCF/KITLG | KITLG | KIT ligand | 1.4589 | 1.7055 | 23.3005 |
| 86 | SCF R/KIT | KIT | V-kit Hardy-Zuckerman 4 feline sarcoma viral oncogene homolog | 0.1207 | 1.0085 | 30.2559 |
| 87 | SDF-1α/CXCL12 | CXCL12 | Chemokine (C-X-C motif) ligand 12 | 1.4895 | 2.2387 | 23.3005 |
| 88 | Serpin A4 | SERPINA4 | Serpin peptidase inhibitor, clade A (alpha-1 antiproteinase, antitrypsin), member 4 | 0.4920 | 1.0403 | 30.2559 |
| 89 | sFRP-3/FRZB | FRZB | Frizzled-related protein | 0.2454 | 0.6650 | 30.2559 |
| 90 | Siglec-5 | SIGLEC5 | Sialic acid binding Ig-like lectin 5 | 1.1791 | 1.4591 | 27.3549 |
| 91 | TACE/ADAM17 | ADAM17 | ADAM metallopeptidase domain 17 | 1.0614 | 139.1607 | 27.3549 |
| 92 | TECK/CCL25 | CCL25 | Chemokine (C-C motif) ligand 25 | 0.5421 | 1.3401 | 30.2559 |
| 93 | Thyroglobulin | TG | Thyroglobulin | 1.0199 | 158.7939 | 27.3549 |
| 94 | TIMP-4 | TIMP4 | TIMP metallopeptidase inhibitor 4 | 0.3559 | 1.0786 | 30.2559 |
| 95 | TNF sRI | TNFRSF1A | Tumor necrosis factor receptor superfamily, member 1A | 0.2323 | 1.4680 | 30.2559 |
| 96 | TNF sRII | TNFRSF1B | Tumor necrosis factor receptor superfamily, member 1B | 1.9127 | 1.4792 | 21.5539 |
| 97 | VEGF | VEGFA | Vascular endothelial growth factor A | 0.2144 | 1.0000 | 30.2559 |
| 98 | VEGF R3/FLT4 | FLT4 | Fms-related tyrosine kinase 4 | 0.3176 | 1.7404 | 30.2559 |
| 99 | VEGF-D/FIGF | FIGF | C-fos induced growth factor (vascular endothelial growth factor D) | 0.1393 | 1.2027 | 30.2559 |
| 100 | WIF-1 | WIF1 | Wnt inhibitory factor 1 | 0.0186 | 1.0347 | 30.2559 |
| 101 | XEDAR | EDA2R | Ectodysplasin A2 receptor | 0.8952 | 1.2100 | 27.9221 |
| 102 | β-NGF | NGF | Nerve growth factor (beta polypeptide) | 0.0188 | 1.3566 | 30.2559 |
| 103 | 2B4/Cd244 | Cd244 | CD244 natural killer cell receptor 2B4 | -1.0947 | 0.5376 | 14.6894 |
| 104 | 4-1BB/TNFRSF9 | TNFRSF9 | Tumor necrosis factor receptor superfamily member 9 | -1.3752 | 0.2716 | 10.2381 |
| 105 | 6Ckine/CCL21 | CCL21 | Chemokine (C-C motif) ligand 21 | -0.2563 | 0.8698 | 21.9388 |
| 106 | Activin A/INHBA | INHBA | Activin A | -0.9518 | 0.8216 | 14.6894 |
| 107 | ADAM9 | ADAM9 | AADAM metallopeptidase domain 9 | -1.0415 | 0.3470 | 14.6894 |
| 108 | AFP | AFP | Alpha-1-fetoprotein | -1.4351 | 0.5222 | 10.2381 |
| 109 | AGRP | AGRP | Agouti related protein homolog (mouse) | -1.5790 | 0.7152 | 8.7755 |
| 110 | ALCAM | ALCAM | Activated leukocyte cell adhesion molecule | -1.7823 | 0.6138 | 8.7755 |
| 111 | ANG-1 | ANGPT1 | Angiopoietin 1 | -1.8111 | 0.6599 | 5.5844 |
| 112 | Angiostatin/PLG | PLG | Plasminogen | -0.9494 | 0.9032 | 14.6894 |
| 113 | ANGPTL4 | ANGPTL4 | Angiopoietin-like 4 | -0.3921 | 0.8127 | 21.9388 |
| 114 | AXL/UFO | AXL | AXL receptor tyrosine kinase | -0.3383 | 1.2656 | 21.9388 |
| 115 | BCMA/TNFRSF17 | TNFRSF17 | Tumor necrosis factor receptor superfamily, member 17 | -1.5568 | 0.6967 | 9.9078 |
| 116 | BDNF | BDNF | Brain-derived neurotrophic factor | -3.1158 | 0.2103 | 0.0000 |
| 117 | BMP-2 | BMP2 | Bone morphogenetic protein 2 | -0.2217 | 0.9701 | 21.9388 |
| 118 | BMP-9/GDF2 | GDF2 | Growth differentiation factor 2 | -0.3951 | 0.9187 | 21.9388 |
| 119 | BTC | BTC | Betacellulin | -0.1813 | 0.4438 | 21.9388 |
| 120 | C5a | C5 | Complement component 5a | -1.2427 | 0.6005 | 12.6007 |
| 121 | Catheprin S/CTSS | CTSS | Cathepsin S | -0.8357 | 0.8704 | 17.9167 |
| 122 | Cathepsin L/CTSL | CTSL | Cathepsin L | -0.4035 | 0.5867 | 21.9388 |
| 123 | CCL28 | CCL28 | Chemokine (C-C motif) ligand 28 | -1.7324 | 0.4433 | 8.7755 |
| 124 | CD40 | CD40 | CD40 molecule, TNF receptor superfamily member 5 | -1.3744 | 0.7102 | 10.2381 |
| 125 | CD14 | CD14 | CD14 molecule | -0.8198 | 0.9155 | 17.9167 |
| 126 | CD30/TNFRSF8 | TNFRSF8 | Tumor necrosis factor receptor superfamily, member 8 | -0.1967 | 0.5932 | 21.9388 |
| 127 | CD40L | CD40LG | Cd40 ligand | -1.8309 | 0.5175 | 5.5844 |
| 128 | CEA | CEACAM5 | Carcinoembryonic antigen-related cell adhesion molecule 5 | -0.2185 | 0.9534 | 21.9388 |
| 129 | Chemerin/RARRES2 | RARRES2 | Retinoic acid receptor responder (tazarotene induced) 2 | -1.6985 | 0.7027 | 8.7755 |
| 130 | Cripto-1/TDGF1 | TDGF1 | Teratocarcinoma-derived growth factor 1 | -1.5710 | 0.7937 | 8.7755 |
| 131 | CXCL16 | CXCL16 | Chemokine (C-X-C motif) ligand 16 | -0.1888 | 0.7921 | 21.9388 |
| 132 | DcR3/TNFRSF6B | TNFRSF6B | Tumor necrosis factor receptor superfamily, member 6b, decoy | -2.3713 | 0.9974 | 3.2331 |
| 133 | DKK-1 | DKK1 | Dickkopf WNT signaling pathway inhibitor 1 | -1.0308 | 0.8624 | 14.6894 |
| 134 | DR6/TNFRSF21 | TNFRSF21 | Tumor necrosis factor receptor superfamily, member 21 | -1.6976 | 0.6765 | 8.7755 |
| 135 | Dtk/TYRO3 | TYRO3 | TYRO3 protein tyrosine kinase | -1.9616 | 0.6915 | 5.5844 |
| 136 | E-Cadherin/CDH1 | CDH1 | Cadherin 1, type 1, E-cadherin (epithelial) | -0.8091 | 0.8665 | 17.9167 |
| 137 | EGF | EGF | Epidermal growth factor | -4.9168 | 0.1362 | 0.0000 |
| 138 | EGF R | EGFR | Epidermal growth factor receptor | -1.7366 | 0.3904 | 8.7755 |
| 139 | EG-VEGF/PROK1 | PROK1 | Prokineticin 1 Growth Factor | -0.3516 | 0.6440 | 21.9388 |
| 140 | ENA-78/CXCL5 | CXCL5 | Chemokine (C-X-C motif) ligand 5 | -2.3350 | 0.2851 | 3.2331 |
| 141 | Endoglin | ENG | Endoglin | -3.5552 | 0.5708 | 0.0000 |
| 142 | Eotaxin-2/CCL24 | CCL24 | Chemokine (C-C motif) ligand 24 | -0.8107 | 0.8254 | 17.9167 |
| 143 | EpCAM | EPCAM | Epithelial cell adhesion molecule | -0.0752 | 0.9068 | 21.9388 |
| 144 | ErbB3 | ERBB3 | V-erb-b2 avian erythroblastic leukemia viral oncogene homolog 3 | -0.2965 | 0.9718 | 21.9388 |
| 145 | E-Selectin/SELE | SELE | Selectin E | -0.2421 | 0.9157 | 21.9388 |
| 146 | FABP2 | FABP2 | Fatty acid binding protein 2, intestinal | -0.1967 | 0.8159 | 21.9388 |
| 147 | FAP | FAP | Fibroblast activation protein, alpha | -0.6707 | 0.9461 | 20.8625 |
| 148 | Fas L | FASLG | Fas ligand (TNF superfamily, member 6) | -1.5471 | 0.7724 | 9.9078 |
| 149 | Fcγ RIIB/C | FCGR2B | Fc fragment of igg, low affinity iib, receptor (CD32) | -1.2131 | 0.8368 | 12.6007 |
| 150 | FGF-19 | FGF19 | Fibroblast growth factor 19 | -0.2351 | 1.6133 | 21.9388 |
| 151 | FGF-4 | FGF4 | Fibroblast growth factor 4 | -1.4865 | 0.8664 | 9.9078 |
| 152 | Flt-3L | FLT3LG | Fms-like tyrosine kinase 3 Ligand | -1.5727 | 0.7022 | 8.7755 |
| 153 | Follistatin/FST | FST | Follistatin | -1.4782 | 0.7485 | 9.9078 |
| 154 | FSH | FSH | Follicle-stimulating hormone | -0.6013 | 0.8405 | 21.9388 |
| 155 | Galectin-7/LGALS7 | LGALS7 | Lectin, galactoside-binding, soluble, 7 | -0.8320 | 0.5774 | 17.9167 |
| 156 | GCP-2/CXCL6 | CXCL6 | Chemokine (C-X-C motif) ligand 6 | -2.0501 | 0.6231 | 5.5844 |
| 157 | G-CSF | CSF3 | Colony stimulating factor 3 (granulocyte) | -1.4422 | 0.4917 | 10.2381 |
| 158 | GM-CSF/CSF2 | CSF2 | Colony stimulating factor 2 (granulocyte-macrophage) | -3.2179 | 0.5874 | 0.0000 |
| 159 | GRO/CXCR2 | CXCR2 | Chemokine (C-X-C motif) receptor 2 | -2.5595 | 0.6606 | 0.0000 |
| 160 | GROα/CXCL1 | CXCL1 | Chemokine (C-X-C motif) ligand 1 (melanoma growth stimulating activity, alpha) | -5.2297 | 0.6251 | 0.0000 |
| 161 | HCC-4/CCL16 | CCL16 | Chemokine (C-C motif) ligand 16 | -0.2188 | 0.8242 | 21.9388 |
| 162 | HCGβ | CGB | Chorionic gonadotropin, beta polypeptide | -0.7295 | 0.4000 | 19.2717 |
| 163 | HGF | HGF | Hepatocyte growth factor (hepapoietin A; scatter factor) | -0.5301 | 0.9133 | 21.9388 |
| 164 | HGF R/MET | MET | Met proto-oncogene | -0.8640 | 0.9287 | 17.9167 |
| 165 | HVEM/TNFRSF14 | TNFRSF14 | Tumor necrosis factor receptor superfamily, member 14 | -1.1025 | 0.7739 | 14.6894 |
| 166 | ICAM-2 | ICAM2 | Intercellular adhesion molecule 2 | -0.0731 | 0.9668 | 21.9388 |
| 167 | ICAM-3 | ICAM3 | Intercellular adhesion molecule 3 | -0.8146 | 0.7246 | 17.9167 |
| 168 | IFNα/β R2 | IFNAR2 | Interferon (alpha, beta and omega) receptor 2 | -1.4886 | 3.3535 | 9.9078 |
| 169 | IFNγ | IFNG | Interferon gamma | -1.5118 | 0.4055 | 9.9078 |
| 170 | IGFBP-1 | IGFBP1 | Insulin-like growth factor binding protein 1 | -0.4841 | 0.6477 | 21.9388 |
| 171 | IGFBP-2 | IGFBP2 | Insulin-like growth factor binding protein 2 | -0.0317 | 0.9957 | 21.9388 |
| 172 | IGFBP-6 | IGFBP6 | Insulin-like growth factor binding protein 6 | -1.8141 | 0.8040 | 5.5844 |
| 173 | IGF-I | IGF1 | Insulin-like growth factor 1 (somatomedin C) | -0.3557 | 2.2879 | 21.9388 |
| 174 | IGF-1 SR | IGF1R | Insulin-like growth factor 1 receptor | -0.1558 | 1.6534 | 21.9388 |
| 175 | IGF-II R | IGF2R | Insulin-like growth factor II receptor | -1.7358 | 0.7246 | 8.7755 |
| 176 | IL-1R6 | IL1RL2 | Interleukin 1 receptor-like 2 | -0.4460 | 0.8761 | 21.9388 |
| 177 | IL-1RL1 | IL1RL1 | Interleukin 1 receptor-like 1 | -0.9937 | 0.8220 | 14.6894 |
| 178 | IL-1 sRII | IL1R2 | Interleukin-1 receptor type II | -0.3770 | 1.4545 | 21.9388 |
| 179 | IL-11 | IL11 | Interleukin 11 | -1.6232 | 0.4739 | 8.7755 |
| 180 | IL-12p70 | IL12A | Interleukin 12A (natural killer cell stimulatory factor 1, cytotoxic lymphocyte maturation factor 1, p35) | -0.3389 | 0.3294 | 21.9388 |
| 181 | IL-13 | IL13 | Interleukin 13 | -1.9424 | 0.2124 | 5.5844 |
| 182 | IL-13 R1 | IL13RA1 | Interleukin 13 receptor, alpha 1 | -1.0869 | 0.8205 | 14.6894 |
| 183 | IL-13R2 | IL13RA2 | Interleukin 13 receptor, alpha 2 | -0.6735 | 0.9345 | 19.2717 |
| 184 | IL-15 | IL15 | Interleukin 15 | -4.9955 | 0.6646 | 0.0000 |
| 185 | IL-16 | IL16 | Interleukin 16 | -3.0149 | 0.5824 | 0.0000 |
| 186 | IL-17 | IL17A | Interleukin 17A | -2.5240 | 0.5301 | 0.0000 |
| 187 | IL-17B | IL17B | Interleukin 17B | -0.9593 | 0.8065 | 14.6894 |
| 188 | IL-17F | IL17F | Interleukin 17F | -1.0225 | 0.1720 | 14.6894 |
| 189 | IL-17R | IL17RA | Interleukin 17 receptor A | -0.0495 | 0.9748 | 21.9388 |
| 190 | IL-18 BPa | IL18BP | Interleukin-18-binding protein | -0.5238 | 1.3573 | 21.9388 |
| 191 | IL-18 Rβ | IL18RAP | Interleukin-18 receptor accessory protein | -0.0219 | 0.5626 | 21.9388 |
| 192 | IL-1ra | IL1RN | Interleukin 1 receptor antagonist | -1.4307 | 0.3919 | 10.2381 |
| 193 | IL-1αa | IL1A | Interleukin 1, alpha | -1.2849 | 0.1102 | 11.6216 |
| 194 | IL-2 | IL2 | Interleukin 2 | -1.6878 | 0.2091 | 8.7755 |
| 195 | IL-2 Rα | IL2RA | Interleukin 2 receptor, alpha | -1.0985 | 0.8452 | 14.6894 |
| 196 | IL-2 Rβ | IL2RB | Interleukin 2 receptor, beta | -1.1085 | 0.8424 | 12.6007 |
| 197 | IL-21 | IL21 | Interleukin 21 | -1.3843 | 0.2191 | 10.2381 |
| 198 | IL-23 | IL23A | Interleukin 23, alpha subunit p19 | -1.5800 | 0.7142 | 8.7755 |
| 199 | IL-24 | IL24 | Interleukin 24 | -0.5783 | 0.9241 | 21.9388 |
| 200 | IL-29 | IFNL1 | Interferon, lambda 1 | -0.8229 | 0.8221 | 17.9167 |
| 201 | IL-3 | IL3 | Interleukin 3 (colony-stimulating factor, multiple) | -1.1639 | 0.9763 | 12.6007 |
| 202 | IL-31 | IL31 | Interleukin 31 | -0.7370 | 0.2821 | 19.2717 |
| 203 | IL-33 | IL33 | Interleukin 33 | -1.4028 | 0.3008 | 10.2381 |
| 204 | IL-4 | IL4 | Interleukin 4 | -1.1140 | 0.3316 | 12.6007 |
| 205 | IL-5 | IL5 | Interleukin 5 | -3.0356 | 0.3828 | 0.0000 |
| 206 | IL-6 | IL6 | Interleukin 6 (interferon, beta 2) | -1.8891 | 0.4795 | 5.5844 |
| 207 | IL-7 | IL7 | Interleukin 7 | -2.9352 | 0.3840 | 0.0000 |
| 208 | IL-8 | IL8 | Interleukin 8 | -3.0268 | 0.1984 | 0.0000 |
| 209 | IL-9 | IL9 | Interleukin 9 | -0.4901 | 2.7551 | 21.9388 |
| 210 | IP-10/CXCL10 | CXCL10 | Chemokine (C-X-C motif) ligand 10 | -2.7947 | 0.6197 | 0.0000 |
| 211 | I-TAC/CXCL11 | CXCL11 | Chemokine (C-X-C motif) ligand 11 | -2.8117 | 0.3635 | 0.0000 |
| 212 | Kallikrein 14 | KLK14 | Kallikrein-related peptidase 14 | -1.0287 | 0.1789 | 14.6894 |
| 213 | LAP/TGFB1 | TGFB1 | Transforming growth factor, beta 1 | -2.3121 | 0.5995 | 3.2331 |
| 214 | LIGHT/TNFSF14 | TNFSF14 | Tumor necrosis factor (ligand) superfamily, member 14 | -0.5019 | 0.5124 | 21.9388 |
| 215 | LIMPII/SCARB2 | SCARB2 | Scavenger receptor class B, member 2 | -0.7147 | 0.3172 | 19.2717 |
| 216 | Lipocalin-2 | LCN2 | Lipocalin 2 | -0.7893 | 0.9253 | 17.9167 |
| 217 | LOX-1/OLR1 | OLR1 | Oxidized low density lipoprotein (lectin-like) receptor 1 | -1.6474 | 0.2790 | 8.7755 |
| 218 | L-Selectin/SELL | Sell | Selectin, lymphocyte | -0.7500 | 0.9079 | 19.2717 |
| 219 | Lymphotactin/XCL1 | XCL1 | Chemokine (C motif) ligand 1 | -0.3279 | 4.4715 | 21.9388 |
| 220 | MCSF R | CSF1R | Colony stimulating factor 1 receptor | -0.4402 | 0.9548 | 21.9388 |
| 221 | MCP-2/CCL8 | CCL8 | Chemokine (C-C motif) ligand 8 | -2.1732 | 0.3747 | 3.2331 |
| 222 | MCP-3/CCL7 | CCL7 | Chemokine (C-C motif) ligand 7 | -0.6983 | 0.8324 | 19.2717 |
| 223 | MIG/CXCL9 | CXCL9 | Chemokine (C-X-C motif) ligand 9 | -0.9906 | 0.7275 | 14.6894 |
| 224 | MIP-1α/CCL3 | CCL3 | Chemokine (C-C motif) ligand 3 | -1.1212 | 0.1633 | 12.6007 |
| 225 | MIP-1β/CCL4 | CCL4 | Chemokine (C-C motif) ligand 4 | -0.5869 | 0.2841 | 21.9388 |
| 226 | MIP-1δ/CCL15 | CCL15 | Chemokine (C-C motif) ligand 15 | -0.5098 | 0.8854 | 21.9388 |
| 227 | MMP-1 | MMP1 | Matrix metallopeptidase 1 (interstitial collagenase) | -0.4511 | 0.5522 | 21.9388 |
| 228 | MMP-3 | MMP3 | Matrix metallopeptidase 3 (stromelysin 1, progelatinase) | -2.2762 | 0.5828 | 3.2331 |
| 229 | MMP-8 | MMP8 | Matrix metallopeptidase 8 (neutrophil collagenase) | -0.8933 | 0.0474 | 17.9167 |
| 230 | NAP-2 | NAP1L4 | Nucleosome assembly protein 1-like 4 | -0.1169 | 1.0175 | 21.9388 |
| 231 | NCAM-1 | NCAM1 | Neural cell adhesion molecule 1 | -0.5112 | 0.9362 | 21.9388 |
| 232 | Neprilysin/MME | MME | Membrane metallo-endopeptidase | -0.0075 | 0.6340 | 21.9388 |
| 233 | NGF R | NGFR | Nerve growth factor receptor | -1.1793 | 0.5535 | 12.6007 |
| 234 | Nidogen-1 | NID1 | Nidogen 1 | -0.4879 | 0.9577 | 21.9388 |
| 235 | NOV | NOV | Nephroblastoma overexpressed | -0.6598 | 0.9347 | 20.8625 |
| 236 | NrCAM | NRCAM | Neuronal cell adhesion molecule | -0.1140 | 0.9207 | 21.9388 |
| 237 | NSE | ENO2 | Enolase 2 (gamma, neuronal) | -3.6160 | 0.4847 | 0.0000 |
| 238 | OPG/TNFRSF11B | TNFRSF11B | Tumor necrosis factor receptor superfamily, member 11b | -1.0804 | 0.7342 | 14.6894 |
| 239 | Osteoactivin | GPNMB | Glycoprotein (transmembrane) nmb | -0.3747 | 0.8413 | 21.9388 |
| 240 | PAI-1 | SERPINE1 | Serpin peptidase inhibitor, clade E (nexin, plasminogen activator inhibitor type 1), member 1 | -1.9161 | 0.7756 | 5.5844 |
| 241 | PD-1/PDCD1 | PDCD1 | Programmed cell death 1 | -0.9988 | 0.3460 | 14.6894 |
| 242 | PDGF Rβ | PDGFRB | Platelet-derived growth factor receptor, beta polypeptide | -2.5427 | 0.3604 | 0.0000 |
| 243 | PDGF-AA | PDGFA | Platelet-derived growth factor alpha polypeptide | -2.4820 | 0.6300 | 0.0000 |
| 244 | PDGF-AB | PDGFAB | Platelet-derived growth factor subunit AB | -2.8786 | 0.5059 | 0.0000 |
| 245 | PDGF-BB | PDGFB | Platelet-derived growth factor beta polypeptide | -2.4513 | 0.5799 | 0.0000 |
| 246 | PECAM-1 | PECAM1 | Platelet endothelial cell adhesion molecule 1 | -3.1185 | 0.4043 | 0.0000 |
| 247 | Prolactin/PRL | PRL | Prolactin | -0.0686 | 0.5411 | 21.9388 |
| 248 | PSA/KLK3 | KLK3 | Kallikrein-related peptidase 3 | -0.5898 | 3.9488 | 21.9388 |
| 249 | Resistin/RETN | RETN | Resistin | -1.0075 | 0.8525 | 14.6894 |
| 250 | SDF-1β/CXCL12B | CXCL12b | Chemokine (C-X-C motif) ligand 12b (stromal cell-derived factor 1) | -1.1599 | 0.8153 | 12.6007 |
| 251 | sgp130/IL6ST | IL6ST | Interleukin 6 signal transducer (gp130, oncostatin M receptor) | -0.6720 | 0.9301 | 20.8625 |
| 252 | Shh N | Shh N | Sonic hedgehog n-terminus | -0.8822 | 0.8950 | 17.9167 |
| 253 | Siglec-9 | SIGLEC9 | Sialic acid-binding Ig-like lectin 9 | -1.2106 | 0.2324 | 12.6007 |
| 254 | TARC/CCL17 | CCL17 | Chemokine (C-C motif) ligand 17 | -2.4505 | 0.1980 | 0.0000 |
| 255 | TGF-β2 | TGFB2 | Transforming growth factor, beta 2 | -0.7320 | 0.9224 | 19.2717 |
| 256 | TGFα | TGFA | Transforming growth factor, alpha | -0.5917 | 0.2672 | 21.9388 |
| 257 | TGFβ1 | TGFB1 | Transforming growth factor, beta 1 | -1.0374 | 0.4589 | 14.6894 |
| 258 | TGFβ3 | TGFB3 | Transforming growth factor, beta 3 | -1.2064 | 0.2351 | 12.6007 |
| 259 | THBD | THBD | Thrombomodulin | -0.7765 | 0.8423 | 17.9167 |
| 260 | Tie-2/TEK | TEK | TEK tyrosine kinase, endothelial | -0.4878 | 0.8845 | 21.9388 |
| 261 | TIM-1/HAVCR1 | HAVCR1 | Hepatitis A virus cellular receptor 1 | -0.1296 | 0.5794 | 21.9388 |
| 262 | TIMP-1 | TIMP1 | TIMP metallopeptidase inhibitor 1 | -0.9284 | 0.9088 | 14.6894 |
| 263 | TIMP-2 | TIMP2 | TIMP metallopeptidase inhibitor 2 | -1.1612 | 0.8072 | 12.6007 |
| 264 | TLR2 | TLR2 | Toll-like receptor 2 | -0.3299 | 0.5072 | 21.9388 |
| 265 | TNFα | TNF | Tumor necrosis factor | -0.7989 | 0.0024 | 17.9167 |
| 266 | TNFβ | LTA | Lymphotoxin alpha | -2.0457 | 0.0180 | 5.5844 |
| 267 | TPO | TPO | Thyroid peroxidase | -0.7127 | 0.9253 | 19.2717 |
| 268 | TRAIL R1 | TNFRSF10A | Tumor necrosis factor receptor superfamily, member 10a | -2.4758 | 0.2591 | 0.0000 |
| 269 | TRAIL R3 | TNFRSF10C | Tumor necrosis factor receptor superfamily, member 10c, decoy without an intracellular domain | -0.9730 | 0.7837 | 14.6894 |
| 270 | TRAIL R4 | TNFRSF10D | Tumor necrosis factor receptor superfamily, member 10d, decoy with truncated death domain | -0.8149 | 0.8574 | 17.9167 |
| 271 | Transferrin/TF | TF | Transferrin | -0.3379 | 0.9503 | 21.9388 |
| 272 | Trappin-2/PI3 | PI3 | Peptidase inhibitor 3, skin-derived | -0.6920 | 0.9297 | 19.2717 |
| 273 | TREM-1 | TREM1 | Triggering receptor expressed on myeloid cells 1 | -0.8083 | 0.9062 | 17.9167 |
| 274 | TSH/CGA | CGA | Glycoprotein hormones, alpha polypeptide | -2.5143 | 0.4286 | 0.0000 |
| 275 | TSLP | TSLP | Thymic stromal lymphopoietin | -0.6305 | 0.1507 | 20.8625 |
| 276 | uPAR | PLAUR | Plasminogen activator, urokinase receptor | -0.3449 | 0.7304 | 21.9388 |
| 277 | VCAM-1 | VCAM1 | Vascular cell adhesion molecule 1 | -0.0789 | 0.9934 | 21.9388 |
| 278 | VEGF R2 | KDR | Kinase insert domain receptor (a type III receptor tyrosine kinase) | -0.2376 | 0.8712 | 21.9388 |
| 279 | VEGF-C | VEGFC | Vascular endothelial growth factor C | -1.2769 | 0.8209 | 11.6216 |
| 280 | VEGF R1/FLT1 | FLT1 | Fms-related tyrosine kinase 1 | -0.6036 | 0.9646 | 21.9388 |

* Fold change between ACS cases and controls.

**Supplementary Table 2. Levels of cytokines in each of the validation populations. ***

| **Cytokines** | **Validation set 1** | | | **Validation set 2** | | | **Nested case-control study** | | |
| --- | --- | --- | --- | --- | --- | --- | --- | --- | --- |
|  | **Controls (n=107)** | **Cases (n=107)** | ***P*^†^** | **Controls** | **Cases** | ***P*** | **Controls (n=318)** | **Cases (n=318)** | ***P*** |
|  |  |  |  | **(n=103)** | **(n=103)** |  |  |  |  |
| Osteopontin (ng/ml) | 58.3 | 82.0 | <0.001 | 50.9 | 125.3 | <0.001 | 50.4 | 54.3 | 0.027 |
|  | (43.6-69.3) | (56.6-139.8) |  | (38.8-64.4) | (76.1-192.7) |  | (38.9-63.4) | (43.6-71.3) |  |
| BDNF (ng/ml) | 6.5 | 2.4 | <0.001 | 3.3 | 2.3 | <0.001 | 21.0 | 19.1 | 0.260 |
|  | (3.7-10.5) | (1.0-6.3) |  | (2.3-7.5) | (0.8-5.1) |  | (13.1-32.0) | (13.6-27.3) |  |
| CCL23 (ng/ml) | 5.2 | 8.7 | <0.001 | 4.0 | 6.3 | <0.001 | 2.2 | 2.3 | 0.995 |
|  | (3.9-7.3) | (5.0-11.0) |  | (1.8-5.3) | (4.8-8.4) |  | (2.0-2.6) | (2.0-2.6) |  |
| MSP (ng/ml) | 89.2 | 95.4 | 0.097 | 97.8 | 118.2 | 0.385 | - | - | - |
|  | (63.4-120.1) | (60.0-148.1) |  | (62.8-165.0) | (70.2-186.7) |  |  |  |  |
| CRP (mg/l) | 0.5 | 4.5 | <0.001 | 0.6 | 6.8 | <0.001 | 1.5 | 2.0 | 0.032 |
|  | (0.2-1.1) | (1.0-21.5) |  | (0.3-1.6) | (2.5-17.6) |  | (0.7-3.6) | (0.9-4.7) |  |

* Plasma cytokines are represented as median (25^th^, 75^th^) and the distribution difference between cases and controls were tested by Wilcoxon rank-sum test.

| **Supplementary Table 3. Association between replicated cytokines (osteopontin and CRP) and incident ACS in different onset time groups in nested case-control study.** | | | | | | |
| --- | --- | --- | --- | --- | --- | --- |
| **Groups** | **N**  **(cases/controls)** | **Age-adjusted** | |  | **Multivariable-adjusted*** | |
|  |  | **Adjusted Odds Ratio (95% CI) ^†^** | ***P*** |  | **Adjusted Odds Ratio (95% CI)** | ***P*** |
| **Osteopontin** |  |  |  |  |  |  |
| **onset time < 0.5 year** | 49/49 | 1.67 (1.01-2.74) | 0.045 |  | 1.78 (0.95-3.35) | 0.073 |
| **onset time 0.5-1 year** | 124/124 | 1.54 (1.13-2.11) | 0.007 |  | 1.53 (1.08-2.16) | 0.017 |
| **onset time ≥ 1 years** | 145/145 | 1.20 (1.04-1.25) | 0.001 |  | 1.18 (1.03-1.22) | 0.001 |
| **CRP** |  |  |  |  |  |  |
| **onset time < 0.5 year** | 49/49 | 1.32 (0.72-2.42) | 0.373 |  | 1.40 (0.64-3.06) | 0.397 |
| **onset time 0.5-1 year** | 124/124 | 1.62 (1.14-2.29) | 0.007 |  | 1.56 (1.04-2.35) | 0.032 |
| **onset time ≥ 1 years** | 145/145 | 1.10 (0.81-1.50) | 0.529 |  | 1.16 (0.81-1.65) | 0.418 |
| * Adjusted for age, BMI, smoking status, total cholesterol, low-density lipoprotein cholesterol, triglycerides, fasting glucose, estimated glomerular filtration rate, systolic blood pressure, anti-hypertensive medication, and lipid-lowing medication. | | | | | | |
| † OR for each SD change of the ln-transformed plasma cytokines. | | | | | |  |

| **Supplementary Table 4. Association of incident ACS with replicated cytokines stratified by established risk factors in the nested case-control study.** | | | | | | | |
| --- | --- | --- | --- | --- | --- | --- | --- |
| **Groups** | **N** | **Tertiles of cytokines** | | | | ***P* for trend *** | ***P* for interaction^†^** |
|  |  | **T1** | **T2** | **T3** | |  |  |
| **Osteopontin** | | | | | | | |
| **Age** |  |  |  |  | |  | 0.894 |
| <65 years | 248 | [ref] | 1.57 (0.78-3.16) | 2.04 (0.97-4.31) | | 0.061 |  |
| ≥65 years | 388 | [ref] | 1.53 (0.85-2.74) | 1.59 (0.92-2.75) | | 0.117 |  |
| **Sex** |  |  |  |  | |  | 0.893 |
| Men | 340 | [ref] | 1.56 (0.83-2.92) | 1.95 (1.05-3.63) | | 0.037 |  |
| Women | 296 | [ref] | 1.76 (0.94-3.30) | 1.81 (0.97-3.36) | | 0.066 |  |
| **Smoking** |  |  |  |  | |  | 0.274 |
| Never-smokers | 378 | [ref] | 1.66 (0.95-2.90) | 2.31 (1.32-4.04) | | 0.004 |  |
| Ever-smokers | 258 | [ref] | 1.49 (0.74-2.99) | 1.63 (0.79-3.39) | | 0.343 |  |
| **BMI** |  |  |  |  | |  | 0.309 |
| < 24 kg/m^2^ | 327 | [ref] | 2.53 (1.18-5.46) | 3.07 (1.37-6.87) | | 0.007 |  |
| ≥24 kg/m^2^ | 309 | [ref] | 1.41 (0.82-2.43) | 1.65 (0.97-2.82) | | 0.067 |  |
| **Hypertension** | |  |  | | | | 0.499 |
| Yes | 389 | [ref] | 1.86 (1.04-3.31) | 2.35 (1.30-4.24) | | 0.006 |  |
| No | 247 | [ref] | 1.63 (0.77-3.47) | 1.70 (0.85-3.39) | | 0.141 |  |
| **Diabetes** |  |  |  |  | |  | 0.800 |
| Yes | 539 | [ref] | 1.72 (1.07-2.77) | 1.84 (1.15-2.95) | | 0.015 |  |
| No | 97 | [ref] | 1.15 (0.33-4.03) | 2.82 (0.71-11.30) | | 0.157 |  |
| **CRP** | | | | | | | |
| **Age** |  |  |  |  | |  | 0.064 |
| <65 years | 248 | [ref] | 1.15 (0.57-2.36) | 1.21 (0.98-1.49) | | 0.498 |  |
| ≥65 years | 388 | [ref] | 1.33 (0.75-2.37) | 2.07 (1.16-3.69) | | 0.012 |  |
| **Sex** |  |  |  |  | |  | 0.085 |
| Men | 340 | [ref] | 1.93 (1.05-3.53) | 3.14 (1.68-5.88) | | 0.001 |  |
| Women | 296 | [ref] | 1.44 (0.71-2.93) | 1.60 (0.83-3.08) | | 0.208 |  |
| **Smoking** |  |  |  |  | |  | 0.090 |
| Never-smokers | 379 | [ref] | 0.75 (0.59-1.86) | 1.05 (0.59-1.86) | | 0.854 |  |
| Ever-smokers | 258 | [ref] | 1.53 (0.76-3.05) | 2.47 (1.23-4.96) | | 0.011 |  |
| **BMI** |  |  |  |  | |  | 0.226 |
| < 24 kg/m^2^ | 327 | [ref] | 1.03 (0.49-2.16) | 1.07 (0.54-2.10) | | 0.924 |  |
| ≥24 kg/m^2^ | 309 | [ref] | 0.93 (0.52-1.67) | 1.66 (0.96-2.88) | | 0.043 |  |
| **Hypertension** | |  |  | |  | | 0.997 |
| Yes | 389 | [ref] | 0.84 (0.49-1.44) | 1.51 (0.87-2.63) | | 0.156 |  |
| No | 247 | [ref] | 1.38 (0.63-3.04) | 1.47 (0.71-3.06) | | 0.331 |  |
| **Diabetes** |  |  |  |  | |  | 0.565 |
| Yes | 539 | [ref] | 0.92 (0.57-1.48) | 1.49 (0.93-2.38) | | 0.087 |  |
| No | 97 | [ref] | 1.70 (0.45-6.39) | 1.54 (0.41-5.79) | | 0.561 |  |
| ORs were adjusted for age, BMI, smoking status, total cholesterol, low-density lipoprotein cholesterol, triglycerides, fasting glucose, estimated glomerular filtration rate, systolic blood pressure, anti-hypertensive medication, and lipid-lowing medication. | | | | | | | |
| * *P* value when we assigned the median value to each tertile and entered this as a continuous variable in the model. | | | | | | | |
| † *P* value for the interaction term of continuous biomarkers*categorical stratifying variable. | | | | | | | |

| **Groups** | **N** | **Before ACS onset** | **After ACS onset** | ***P* ^*^** |
| --- | --- | --- | --- | --- |
| **Osteopontin (ng/ml)** | | | | |
| **ACS** | 82 | 52.6 (38.1-66.3) | 67.8 (54.8-94.7) | <0.001 |
| **Subtypes** | | | | |
| UAP | 73 | 52.4 (40.5-64.9) | 65.8 (53.7-90.5) | <0.001 |
| USTEMI | 7 | 52.8 (39.6-70.7) | 142.9 (69.6-288.9) | 0.018 |
| STEMI | 2 | - (70.6-74.4) | - (50.4-104.4) | 0.655 |
| **Onset time window** | | | | |
| <0.5 year | 2 | - (27.2-44.3) | - (100.6-110.9) | <0.001 |
| 0.5-1 year | 33 | 52.4 (38.9-63.9) | 68.3 (53.6-120.5) | <0.001 |
| ≥1 years | 47 | 52.4 (38.1-61.7) | 66.8 (54.9-78.3) | 0.001 |
| **Stenotic vessels** | | | | |
| 1-VD | 40 | 44.6 (35.5-53.3) | 60.8 (48.5-84.3) | <0.001 |
| 2-VD | 15 | 59.7 (41.9-69.4) | 67.4 (62.9-88.1) | 0.009 |
| 3-VD | 27 | 69.8 (52.8-80.1) | 73.7 (58.7-120.5) | 0.007 |
| **CRP (mg/l)** | | | | |
| **ACS** | 82 | 2.1 (0.9-4.8) | 2.8 (1.4-6.4) | 0.003 |
| **Subtypes** | | | | |
| UAP | 73 | 2.1 (0.9-5.1) | 2.3 (1.4-6.0) | 0.024 |
| USTEMI | 7 | 2.4 (1.6-6.2) | 6.5 (4.8-20.2) | 0.028 |
| STEMI | 2 | - (0.4-0.7) | - (0.4-0.8) | 0.180 |
| **Onset time window** | | | | |
| <0.5 year | 2 | - (1.2-1.6) | - (1.6-1.6) | 0.655 |
| 0.5-1 year | 33 | 2.1 (0.9-4.6) | 2.0 (1.4-5.4) | 0.075 |
| ≥1 years | 47 | 2.2 (0.9-5.8) | 3.7 (1.5-9.4) | 0.016 |
| **Stenotic vessels** | | | | |
| 1-VD | 40 | 1.9 (0.9-4.6) | 2.4 (1.3-4.3) | 0.180 |
| 2-VD | 15 | 2.6 (1.3-4.7) | 1.9 (1.6-9.4) | 0.036 |
| 3-VD | 27 | 2.1 (0.9-5.2) | 2.7 (0.8-6.6) | 0.301 |

**Supplementary Table 5. Plasma osteopontin and CRP levels in different subtypes, onset time groups, and stenotic vessels before and after ACS onset.**

* Plasma osteopontin and CRP levels are represented as median (25^th^, 75^th^) and the distribution differences before and after onset were tested by Wilcoxon rank-sum test.
